# A low-cost uniaxial cell stretcher for six parallel wells

**DOI:** 10.1016/j.ohx.2020.e00162

**Published:** 2020-12-09

**Authors:** Delf Kah, Alexander Winterl, Magdalena Přechová, Ulrike Schöler, Werner Schneider, Oliver Friedrich, Martin Gregor, Ben Fabry

**Affiliations:** a Biophysics Group, Department of Physics, Friedrich-Alexander University Erlangen-Nürnberg (FAU), Erlangen, Germany; b Laboratory of Integrative Biology, Institute of Molecular Genetics of the Czech Academy of Sciences, Prague, Czech Republic; c Institute of Medical Biotechnology, Department of Chemical and Biological Engineering, FAU, Erlangen, Germany; d School in Advanced Optical Technologies, FAU, Erlangen, Germany

**Keywords:** Cell stretcher, 3D printer, Mechanosensing, Cell Mechanics, Uniaxial stretch, YAP

## Abstract

Cells in the lungs, the heart, and numerous other organs, are constantly exposed to dynamic forces and deformations. To mimic these dynamic mechanical loading conditions and to study the resulting cellular responses such as morphological changes or the activation of biochemical signaling pathways, cells are typically seeded on flexible 2D substrates that are uniaxially or biaxially stretched. Here, we present an open-source cell stretcher built from parts of an Anet A8 3D printer. The cell stretcher is controlled by a fully programmable open-source software using GCode and Python. Up to six flexible optically clear substrates can be stretched simultaneously, allowing for comparative multi-batch biological studies including microscopic image analysis. The cell yield from the cell culture area of 4 cm^2^ per substrate is sufficient for Western-blot protein analysis. As a proof-of-concept, we study the activation of the Yes-associated protein (YAP) mechanotransduction pathway in response to increased cytoskeletal tension induced by uniaxial stretching of epithelial cells. Our data support the previously observed activation of the YAP transcription pathway by stretch-induced increase in cytoskeletal tension and demonstrate the suitability of the cell stretcher to study complex mechano-biological processes.

## Graphical abstract

**Figure:**
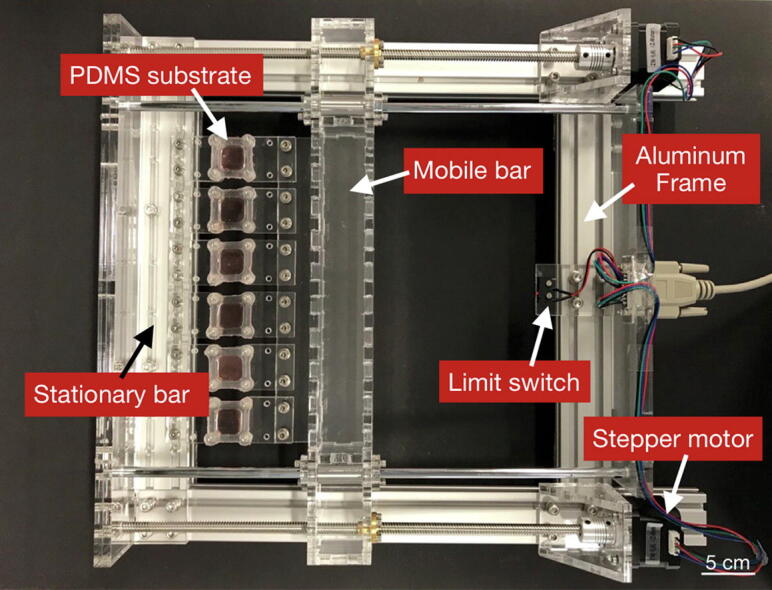

**Specifications table**

**Table:** 

Hardware name	Six-well cell stretcher
Subject area	• Biophysics • Biomedical Engineering • Cell mechanics • Cell biology • Biochemistry • Medicine
Hardware type	Device to apply uniaxial stretch on single cells seeded on flexible PDMS substrates
Open Source License	CC-BY 4.0
Cost of Hardware	~ 375€
Source File Repository	https://doi.org/10.17632/mgkbmytx63.1

## 1 Hardware in context

Research over the last two decades has shown that mechanical signals and their cellular processing (mechanotransduction) are essential for physiological cell behavior, and that disturbed mechanotransduction pathways can lead to a large range of pathological derangements [1], [2], [3], [4]. The influence of mechanical stress on cells is of interest for various scientific fields and can be studied on several levels, ranging e.g. from morphological changes such as cell orientation [5], [6] and the role of cytoskeletal adaptation [7], [8] to the recent identification of mechanosensory ion channels of the piezo family, which are candidates for the direct conversion of stress into chemical signals [9].

Regardless of the specific question, easy-to-use devices are needed to study the influence of mechanical stimulation on cells under laboratory conditions. One of the most widespread devices for this purpose are uniaxial cell stretchers, ranging from home-built devices [5], [10], [11] to commercial systems (reviewed in [12]). Uniaxial cell stretchers typically use linear stepper motors that are connected to a flexible substrate, which is typically made of transparent and highly flexible polydimethylsiloxane (PDMS) polymers (such as Sylgard™184) [13]. Various protocols have been developed to functionalize the PDMS surface, e.g. by coating the surface with the photoactivatable crosslinker Sulfo-SANPAH, which covalently binds to primary amino groups and can therefore be used as a crosslinker between various extracellular matrix (ECM) proteins or peptides and the PDMS surface [14], [15], [16].

Commercially available cell stretchers may cost tens of thousands of USD and are often based on proprietary cell substrates and control software, which further increases operating costs and restricts customization options. Previous publications have presented designs for self-made cell stretchers built from 3D printer parts [17], acrylic glass components [18], and even LEGO bricks [19], but these systems do not allow parallel processing of several samples. Very recently, a parallel cell stretching system for up to six stretchable substrates has been developed and used in a study on the mechano-responsiveness of tendon-associated fibroblasts, but this publication did not provide essential design details of the stretcher [20]).

Here we present an open-source cell stretcher for comparative biological studies, which can impose large-amplitude dynamic stretch on up to six samples in parallel. Our device can be reproduced with limited equipment and technical knowledge for less than 400€. The essential components such as the stepper motor, electronic micro-controller, threaded spindle rods, and bearings are taken from the popular *Anet A8* 3D printer and are complemented by custom acrylic glass components. Acrylic glass is also used to make molds for PDMS cell substrates. Serial communication between the stretcher and a computer is implemented via the established and well documented *GCode* architecture. In addition, we provide scripts in the open-source programming language *Python* [21] that execute typical protocols such as static or cyclic stretch, but can also be easily modified for individual applications.

## 2 Hardware description

The six-well cell stretcher is designed from parts of the *Anet A8* 3D printer and customized acrylic glass parts. The stretcher frame is made from commercially available aluminum profiles. After assembly, the stretcher has dimensions of 47 ✕ 42 ✕ 11 cm (w ✕ d ✕ h) and thus fits into most standard laboratory incubators for long-term experiments. In our experience, the device can be operated long-term under cell culture conditions (37 °C, 95% rel. humidity) without problems.

The following tools are needed to build the six-well cell stretcher:

• A **laser cutting device** for acrylic glass (of up to 6 mm thickness). If such a device is not available in-house, it can be usually found in publicly accessible workshops e.g. of the FabLab network (see www.fablabs.io for locations world-wide), or acrylic parts can be ordered ready-cut via online services. The CAD design files needed for cutting the acrylic glass parts (Suppl. Materials Acryl3MM.pdf and Acryl6MM.pdf) are available under an open BY-SA 4.0 license.
• A **UV light source** for curing acrylic adhesive
• A **soldering iron**
• **Metric plug taps** (M2.5, M3, M4, M5, M6)
• A laboratory heating/drying **oven** (65 °C) for curing the PDMS substrates
• A **3D printer** to fabricate parts of the substrate molds (and stretching clamps)
• Standard tools including a pair of scissors, wire stripping tool, scalpel, caliper, screwdrivers, and Allen keys.

In the following section, we briefly describe the hardware of the six-well cell stretcher, which consists of three units: 1) the stretcher unit including stepper motors and mechanical parts, 2) the electronics and controller unit for operating the stretcher, and 3) the molds for fabricating elastic cell substrates from PDMS.

### 2.1 Stretcher unit

**Fig. 1:**
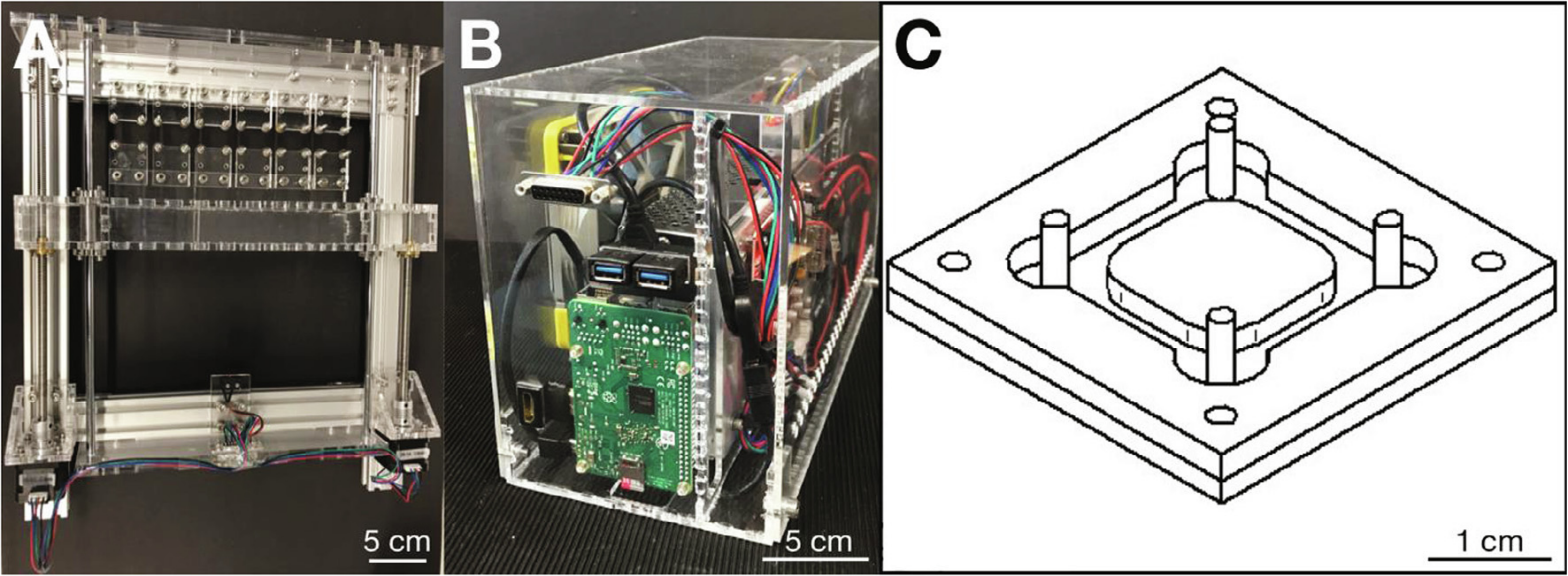
**Hardware.** A: Stretcher unit. B: Electronics and controller unit. C: Technical drawing of the casting mold for PDMS substrates.

Uniaxial stretch is realized by two spindle-driven linear actuators from the Z-axis drive of an *Anet A8* 3D printer, operated by two linear stepper motors (Fig. 1A). The two linear actuators are connected at the moving ends by a mobile bar, or carriage, which is fabricated from 6 mm thick acrylic glass. Up to six elastic cell culture substrates made from PDMS are mounted via hooks between the mobile bar and a stationary bar. The frame of the cell stretcher consists of four aluminum profiles, which are assembled with screws and T-nut bolts. The stepper motors and the stationary bar are attached to the aluminum frame with acrylic glass holders.

For soft PDMS substrates and stretching amplitudes up to 5 mm, load transfer with four hooks (two on each side of the mobile and the stationary bar) results in a deformation of the substrates with a largely reduced amplitude compared to the target stretch (see Section 7). Stretch fidelity can be greatly improved by clamping the two ends of the flexible wells to the stationary and the mobile bar. To fabricate those clamps with a 3D printer, we provide design files (Supl. Material Clamp.stl). Note that for long-term experiments, the clamps should be coated with vaseline to seal the space between the clamps and the substrate surface to prevent loss of culture medium due to capillary effects.

A limit-switch is mounted at the end of the rail (at the maximum stretch position), to prevent the mobile bar from hitting the frame border. The limit-switch is also used to calibrate the zero-stretch position of the stepper motors.

### 2.2 Controller unit

The stretcher unit is connected to the electronics and controller unit via a 15-pole DSUB cable. The electronics and controller unit holds all electrical components (the power supply, and the microcontroller board with the stepper motor drivers) for operating the six-well cell stretcher in a housing made from 3 mm thick acrylic glass (Fig. 1B). The microcontroller board from the *Anet A8* 3D printer receives and processes G-code commands from an external computer or from internal memory, and receives the signals from the limit-switch.

To send G-code commands to the microcontroller board, we provide a computer program with graphical user interface (*pyStretch*), which is installed on a *Raspberry Pi* computer (see Section S1.3). In particular, in Suppl. Materials Stretcher.zip, we provide a .zip file with the open *Raspbian* operating system (Raspbian Buster 2020-02-13), which, when installed, will also install all libraries required to run the *pyStretch* program. Two of the *Raspberry Pi*’s USB ports and one HDMI port can be accessed from outside via angled adapters for connecting peripheral devices (keyboard, mouse, memory stick etc.).

The *Anet A8* power supply (+12 V) is used to power the microcontroller board, the *Raspberry Pi* (via a +12 V to +5 V DC/DC converter), and a small fan, which is mounted to the housing for active cooling of the electronics. As a safety measure, we added a surge-protected power socket and two 2 A micro fuses for connecting the power supply to the 110/220 V power line. It is possible to access the *Raspberry Pi*’s memory card through a hole in the bottom of the housing without opening the cover.

### 2.3 Molds

The design of the elastic PDMS cell substrates was adapted from a previously published system [22]. The mold for fabricating cell substrates consists of two parts: 1) a 3 mm acrylic glass base with 4 posts (for the hooks or clamps) and a raised central platform (for later holding the cell culture medium), and 2) a 3D printed frame (Fig. 1C), the height of which determines the bottom thickness of the PDMS cell substrate. For imaging with an inverted microscope, the bottom thickness of the substrate should not exceed 1 mm to minimize loss of contrast due the high refractive index of PDMS (1.41). We provide a 3D print design file for a mold frame with a height of 3.5 mm, which results in a bottom thickness of 0.5 mm. The design file can be easily adapted to produce frames of varying thickness. Since after molding, the PDMS cell substrates need to be cured at 65 °C for 24 h, we recommend making several molds for simultaneous fabrication of substrates.

## 3 Design files

All design files are under an open CC-BY 4.0 license and can be accessed in a repository under https://doi.org/10.17632/mgkbmytx63.1.

**Design Files Summary**

**Table:** 

Design file name	File type	Open source license	Location of the file
Acryl3MM	SVG and PDF	CC-BY 4.0	https://doi.org/10.17632/mgkbmytx63.1
Acryl6MM	SVG and PDF	CC-BY 4.0	https://doi.org/10.17632/mgkbmytx63.1
MoldFrame	STL	CC-BY 4.0	https://doi.org/10.17632/mgkbmytx63.1
Clamp	STL	CC-BY 4.0	https://doi.org/10.17632/mgkbmytx63.1
Stretcher	ZIP	CC-BY 4.0	https://doi.org/10.17632/mgkbmytx63.1
Raspberry_Pi_Imager_v1.2	ZIP	Apache License, Version 2.0	https://doi.org/10.17632/mgkbmytx63.1
Skynet_for_Stretcher	ZIP	GNU GPL v3	https://doi.org/10.17632/mgkbmytx63.1
Arduino-1.8.0	ZIP	GNU GPL v3	https://doi.org/10.17632/mgkbmytx63.1

• Acryl3MM.pdf: Ready-to-cut file for the acrylic glass parts needed to build the electronics and controller unit housing and the cell substrate molds (Fig. S3).
• Acryl6MM.pdf: Ready-to-cut file for the acrylic glass parts needed to build the stretcher unit (Fig. S2).
• MoldFrame.stl: Ready-to-print file for the 3D printed mold frame (Fig. 1C).
• Clamp.stl: Ready-to-print file for the 3D printed stretching clamps.
• Stretcher.zip: Customized version of the *Raspbian* operating version for the *Raspberry Pi*.
• Raspberry_Pi_Imager_v1.2.zip: Open-source software for Windows PCs to install the *Raspberry Pi* operating system on a memory card.
• Skynet_for_Stretcher.zip: Modified version of the *SkyNet3D* (*Marlin*) open-source firmware for *Anet* 3D printers.
• Arduino-1.8.0.zip: Modified version of the Arduino integrated development environment for Windows PCs with a pre-installed driver for uploading software to the ANET A8 mainboard.

## 4 Bill of materials

The following table itemizes the costs of all materials required to purchase and assemble the stretcher and control unit. Costs for tools, PDMS, and optional components are not listed. Also, the costs for a computer screen, keyboard and mouse, which are required to operate the cell stretcher, are not listed.

**Bill of Materials**

**Table:** 

Designator	Component	Number	Cost per unit (€)	Total cost (€)	Source of materials	Material type
Stretcher	Anet A8 3D printer	1	120.55	120.55	eBay^1^	Other
Stretcher	Aluminum profile 20x40 B-Type Nut 6 310 mm length	2	3.20	6.40	DOLD^2^	Metal
Stretcher	Aluminum profile 20x40 B-Type Nut 6 440 mm length	2	4.43	8.86	DOLD^2^	Metal
Stretcher	Acrylic glass 6 mm × 60 cm × 30 cm	2	12,19	24.38	Plattenzuschnitt24^3^	Polymer
Stretcher	D-SUB connector male (15 Pins)	1	0.24	0.24	Conrad^4^	Electronics
Stretcher	Rubber feet M5 × 15 mm Set (10 pieces)	1	17.55	17.55	Amazon^5^	Polymer
Stretcher	Slim T-slot nuts M5 10 mm × 6 mm (100 pieces)	1	8.99	8.99	Amazon^5^	Metal
Controller	Acrylic glass 3 mm × 60 cm × 30 cm	2	6.58	13.16	Plattenzuschnitt24^3^	Polymer
Controller	D-SUB connector female (15 Pins)	1	0.34	0.34	Conrad^4^	Electronics
Controller	Raspberry Pi 3B	1	36.60	36.60	Amazon^5^	Electronics
Controller	MicroSD Memory Card (16 GB)	1	4.69	4.69	Amazon^5^	Electronics
Controller	USB angle adapter DOWN (CentBest, set of 2 pieces)	1	8.99	8.99	Amazon^5^	Electronics
Controller	HDMI 90° angle (Ubest, set of 3 pieces)	1	4.99	4.99	Amazon^5^	Electronics
Controller	DC/DC Converter 12 V to 5 V (JZK)	1	7.42	7.42	Amazon^5^	Electronics
Controller	Surge-protected power socket (Schaffner FN 282-4-06)	1	20.13	20.13	Conrad^4^	Electronics
Controller	Micro Fuse 2A	2	0.19	0.38	Conrad^4^	Electronics
Controller	Fan (Noctua NF-S12B redux-700)	1	13.90	13.90	Amazon^5^	Electronics
Controller	Anti-vibration pads (Noctua NA-SAVP1, set of 16 pieces)	1	6.90	6.90	Amazon^5^	Polymer
Controller	Micro USB to USB (Type A, male) angled adapter DOWN (~30 cm)	1	6.89	6.89	Amazon^5^	Electronics
Controller	15 pin DSUB cable	1	8.23	8.23	Conrad^4^	Electronics
Controller	IEC power cable	1	2.76	2.76	Conrad^4^	Electronics
General	Screw M2.5 × 16 mm	4	0.04	0.16	DOLD^2^	Metal
General	Screw M2.5 × 10 mm	2	0.04	0.08	DOLD^2^	Metal
General	Screw M3 × 8 mm	16	0.04	0.64	DOLD^2^	Metal
General	Screw M3 × 10 mm	4	0.04	0.16	DOLD^2^	Metal
General	Screw M3 × 12 mm	40	0.04	1.60	DOLD^2^	Metal
General	Screw M4 × 12 mm	24	0.06	1.44	DOLD^2^	Metal
General	Screw M5 × 12 mm	50	0.10	5.00	DOLD^2^	Metal
General	Screw M5 × 20 mm	8	0.20	1.60	DOLD^2^	Metal
General	Screw M6 × 20 mm	4	0.30	1.20	DOLD^2^	Metal
General	Washer M2.5	4	0.12	0.48	DOLD^2^	Metal
General	Washer M3	48	0.12	5.76	DOLD^2^	Metal
General	Washer M4	24	0.14	3.36	DOLD^2^	Metal
General	Washer M5	62	0.13	8.06	DOLD^2^	Metal
General	Washer M6	4	0.16	0.64	DOLD^2^	Metal
General	Spring Washer M2.5	4	0.10	0.40	DOLD^2^	Metal
General	Spring Washer M3	52	0.08	4.16	DOLD^2^	Metal
General	Spring Washer M4	24	0.08	1.92	DOLD^2^	Metal
General	Spring Washer M5	62	0.08	4.96	DOLD^2^	Metal
General	Spring Washer M6	4	0.10	0.40	DOLD^2^	Metal
General	D-SUB screw logs	4	0.65	2.60	Conrad^4^	Metal
General	Acrifix 192 acrylic glass adhesive	1	9.09	9.09	Conrad^4^	Other
**Total**						
	**Stretcher**			186.97€		
	**Controller**			135.38€		
	**General**			53.71€		
	**Sum**			**376.06€**		

^1^ www.ebay.de

^2^ www.dold-mechatronik.de

^3^ www.plattenzuschnitt24.de

^4^ www.conrad.de

^5^ www.amazon.de

## 5 Build Instructions

The supplementary materials include a detailed step-by-step guide for building and assembling the six-well cell stretcher, with lists for the necessary tools and materials and photographs of the device at different stages of building/assembly. Instructions on how to cut acrylic glass sheets or how to operate a 3D printer are not included. In the following section, we give a brief summary of the building process.

### 5.1 Stretcher unit

**Fig. 2:**
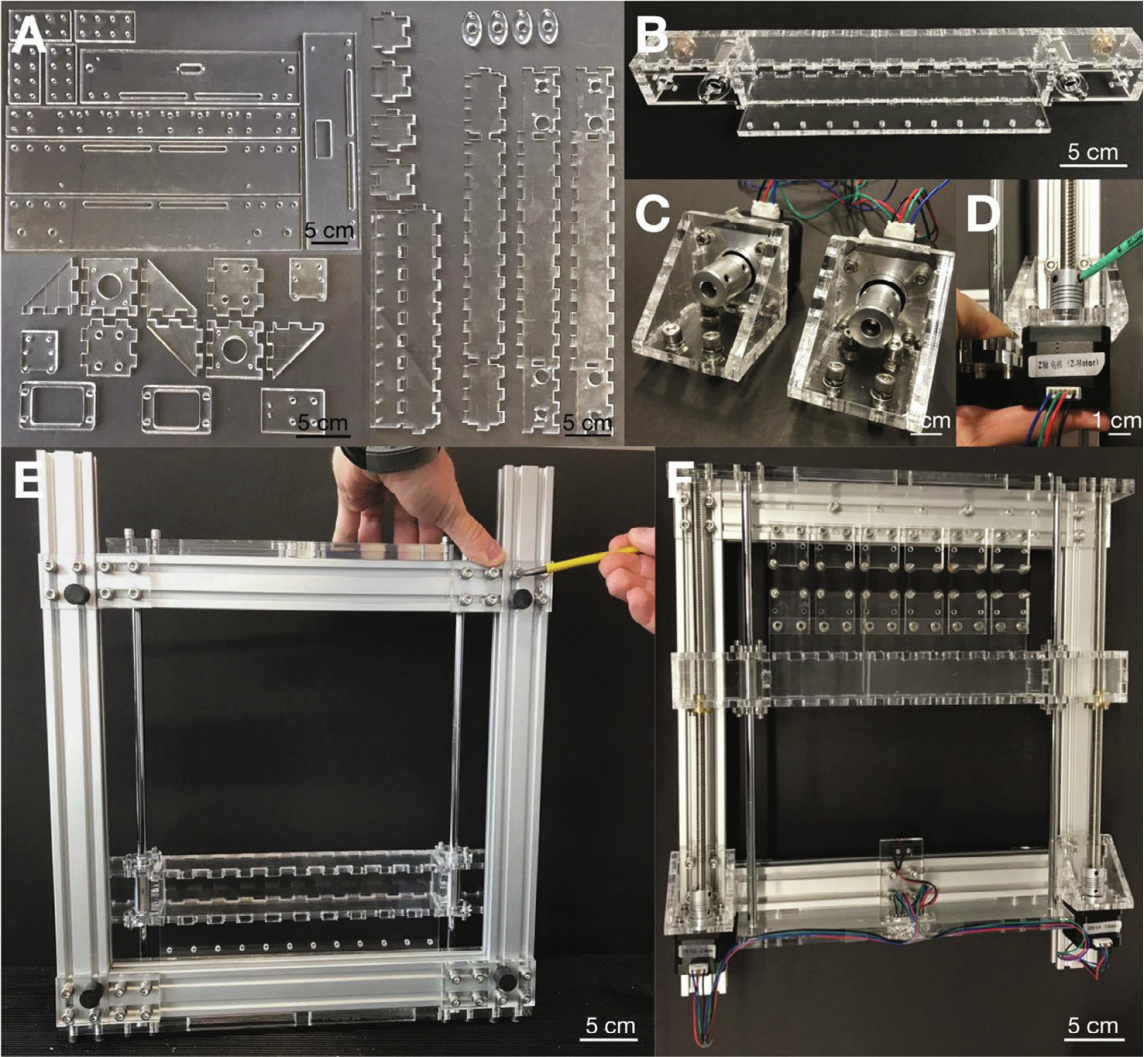
**Assembly of the stretcher unit.** A: Acrylic glass parts (of 6 mm thickness) needed for the stretcher unit. B: Completely assembled carriage with nuts and bearings from the *Anet A8* kit. C: Stepper motors in acrylic glass mounting frames. D: Mounting of a lead screw and motor. E: Assembly of the aluminum frame (bottom view). F: Top view of the final stretcher unit.

Fabricate the acrylic glass parts from two sheets with a size of 6 mm × 30 cm × 60 cm using a laser cutting device (Fig. 2A). Cut threads into the acrylic glass parts according to Fig. S2. Use acrylic adhesive to assemble the carriage bar (Fig. 2B) and mounting frames for the two stepper motors (Fig. 2C). While the acrylic adhesive sets under UV light for 20 min, secure the parts with clamps, adhesive tape etc. Assemble the stretcher frame from aluminum profiles using screws and T-bolt nuts. Arrange two long and one short aluminum profiles in a U-shape and loosely tighten the screws of the middle short profile such that it can still be moved between the adjacent profiles. Attach acrylic end-pieces to both short aluminum profiles orthogonally to the frame. Stick the two lead screws and the two guide rods into the carriage bar and connect one end of the lead screws to the stepper motors (Fig. 2D). Insert both ends of the guide rods as well as the remaining ends of the lead screws into the acrylic end-pieces and tighten the screws of the remaining short aluminum frame while pushing it towards the already fixed end (Fig. 2E). Finally, attach rubber feet to the bottom of the frame, attach the mountings for the elastic cell substrates, and attach the limit switch on the acrylic glass bar. Solder all cables from the stepper motors and the limit switch to a DSUB connector (Fig. 2F).

### 5.2 Electronics and controller unit

**Fig. 3:**
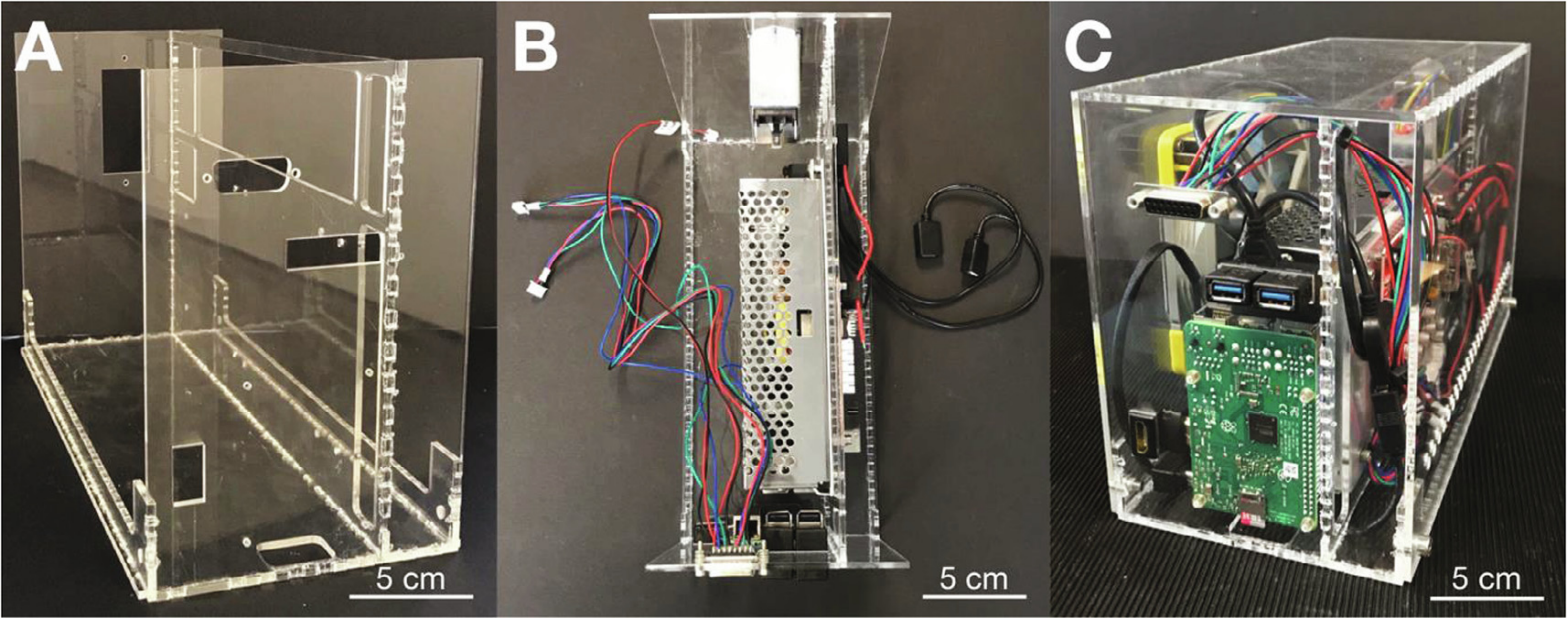
**Assembly of the electronics and controller unit.** A: Acrylic housing without the electronics installed. B: Top view of all parts installed, but unwired. C: Completely assembled controller unit.

Fabricate the acrylic glass parts from two sheets with a size of 3 mm × 30 cm × 60 cm using a laser cutting device. Cut threads into the acrylic glass parts according to Fig. S3. Use acrylic adhesive to assemble the housing of the electronics and controller unit (Fig. 3A). Next, assemble all electrical components in the housing (Fig. 3B): the microcontroller board and power supply from the *Anet A8* kit, a *Raspberry Pi* with two USB and one HDMI angle adapters, a DC/DC converter to supply the *Raspberry Pi* with power, the DSUB connector, and a surge-protected power socket. Mount the fan to the housing cover. Solder the connection cables for the stepper motors and the limit-switch to the DSUB connector and plug the other end into the microcontroller board. Connect the input of the power supply with the surge-protected power socket and the 12 V outputs (as well as GND connections) to the microcontroller board, the fan, and the DC/DC converter. Connect one USB port of the *Raspberry Pi* to the microcontroller board and the microUSB port to the DC/DC connector for power supply. The assembled controller unit with closed cover is shown in Fig. 3C.

### 5.3 Molds

**Fig. 4:**
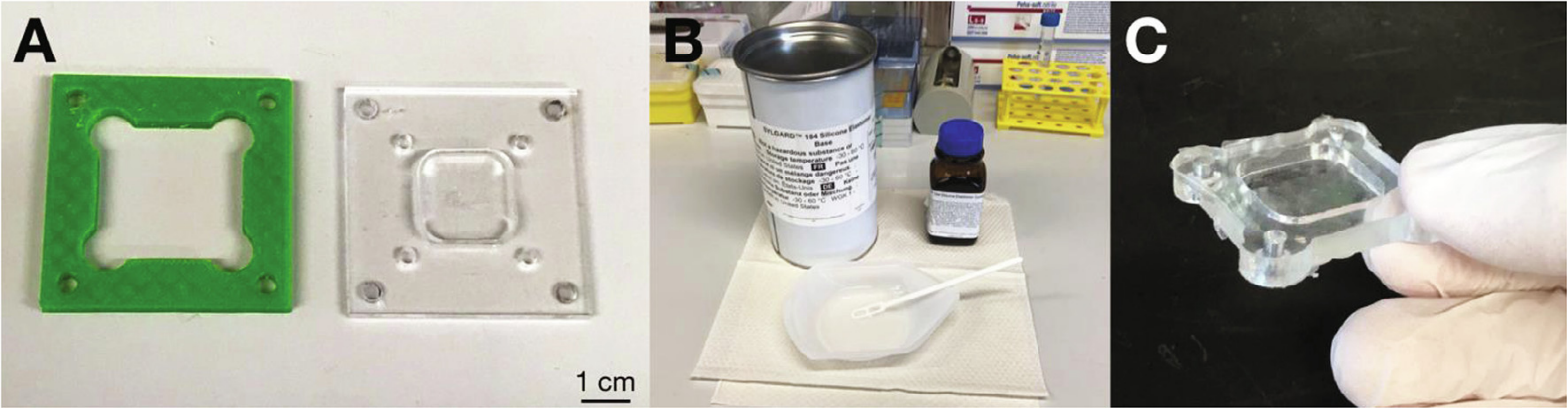
**Fabricating PDMS cell substrates.** A: Mold parts. B: PDMS mixed from base and crosslinker. C: Cell substrate after curing and removal from the mold.

The molds for fabricating elastic PDMS cell substrates consist of two parts: a 3D printed frame and a base with a raised platform, which is fabricated from 3 mm acrylic glass parts (Fig. 4A). Connect the two parts with M4 screws and stick plastic straws with a diameter of 3 mm where later the substrate will have holes for connecting to the stretcher via hooks. Mix PDMS base component and crosslinker (Fig. 4B). The mixing ratio determines the substrate stiffness after curing (a higher crosslinker concentration increases the stiffness) [23]. Table 1 gives an overview of the Young’s moduli for PDMS with different crosslinker-to-base ratios as derived from indentation experiments with a flat cylindrical punch (method described in [24], [25]). With crosslinker-to-base ratios of 1:32 to 1:10, it is possible to tune the substrate stiffness from approximately 100 kPa to 2.4 MPa when curing the PDMS at 65 °C for 6–24 h. Note that the use of PDMS substrates with a lower Young's modulus than 100 kPa may result in altered cell spreading [26].

**Table 1:** **Young’s moduli of PDMS substrates with different crosslinker-to-base ratios.** Samples of approximately 7 g PDMS were prepared in standard 35 mm cell culture dishes and cured for 24 h at 65 °C (except the sample “1:32 6 h”, which was cured for 6 h). Stiffness values are given as mean ± SE derived from 10 measurements per sample.

Crosslinker-to-base ratio (w/w)	Young’s modulus (kPa)
1:10	2391 ± 95
1:12	1522 ± 44
1:14	938 ± 26
1:16	822 ± 15
1:18	744 ± 14
1:20	578 ± 12
1:22	447 ± 8
1:24	382 ± 7
1:26	316 ± 6
1:28	230 ± 6
1:30	189 ± 7
1:32	163 ± 6
1:32 6 h	114 ± 5

To fabricate the stretchable substrates, pour the mixed PDMS into the mold and evacuate the sample for 20 min in a desiccator at 30 mbar if too many air bubbles form during mixing or pouring. Then let the PDMS cure in an oven for 24 h at 65 °C. Afterwards, unmount the frame and carefully remove the cured PDMS substrate from the stamp (Fig. 4C). For this purpose we recommend immersing the molds and substrates in isopropanol. Both the PDMS substrates and the casting molds can be sterilized with standard alcohols for laboratory use (e.g. ethanol or a 70% isopropanol solution).

## 6 Operation Instructions

### 6.1 Sample preparation

i) Fabricate PDMS cell substrates as described in the previous section.
ii) Functionalize the cell substrates by completely wetting the substrate surface with 500 µL of Sulfo-SANPAH at a concentration of 0.5 mg/mL diluted in DPBS. Activate the Sulfo-SANPAH crosslinker through exposure to UV light for 5 min. If successful, the solution changes its color from orange to a dark red. Thoroughly wash out the Sulfo-SANPAH (3 × 5 min) using DPBS.
iii) Coat the cell substrate with an extracellular matrix protein for proper cell adhesion (e.g. 50 µg/mL of collagen or fibronectin diluted in DPBS). Incubate for 2 h at 37 °C (the coating time may vary for different proteins and concentrations), then wash 2x with DBPS.
iv) Add 0.5–2 mL of cell suspension and incubate the sample overnight in a cell culture incubator.

### 6.2 Software description

**Fig. 5:**
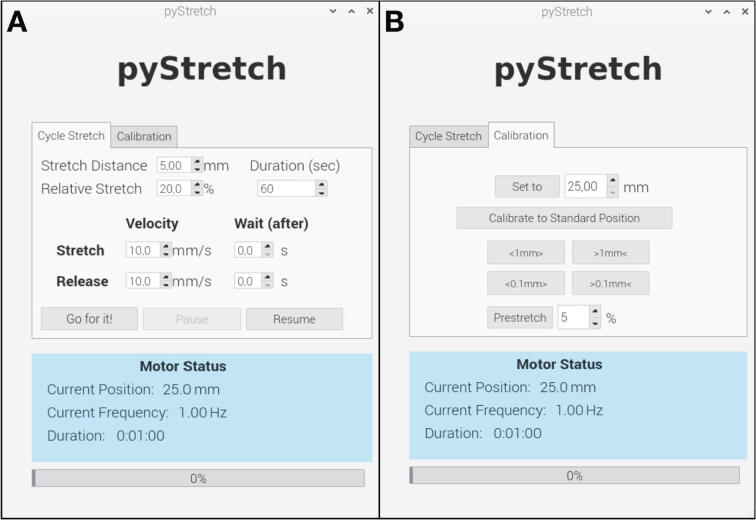
**Software for operation of the cell stretcher.** A: *Cycle Stretch* window for setting up and starting a cyclic stretch protocol. B: *Calibration* window for moving the stretcher to absolute or relative positions and performing a zero-point calibration.

The six-well cell stretcher is operated with a software (*pyStretch*), which has a graphical user interface (GUI) for user-friendly operation. The software is written in the open programming language *Python*. All functions that are needed for serial communication with the *Anet A8* microcontroller board are stored in a separate file (*motor_functions.py*). The GUI of *pyStretch* consists of two windows: in the *Cycle Stretch* window, the user can set the amplitude, duration, speed in stretch and release direction, as well as waiting periods for a cyclic uniaxial stretch (Fig. 5A). In the *Calibration* window, the user can start one-time immediate stretcher movements to absolute and relative distances and perform a zero-point calibration of the stretcher (Fig. 5B). Supplementary Video 1 demonstrates the usage of *pyStretch*.

### 6.3 Stretching for cell biological studies

**Fig. 6:**
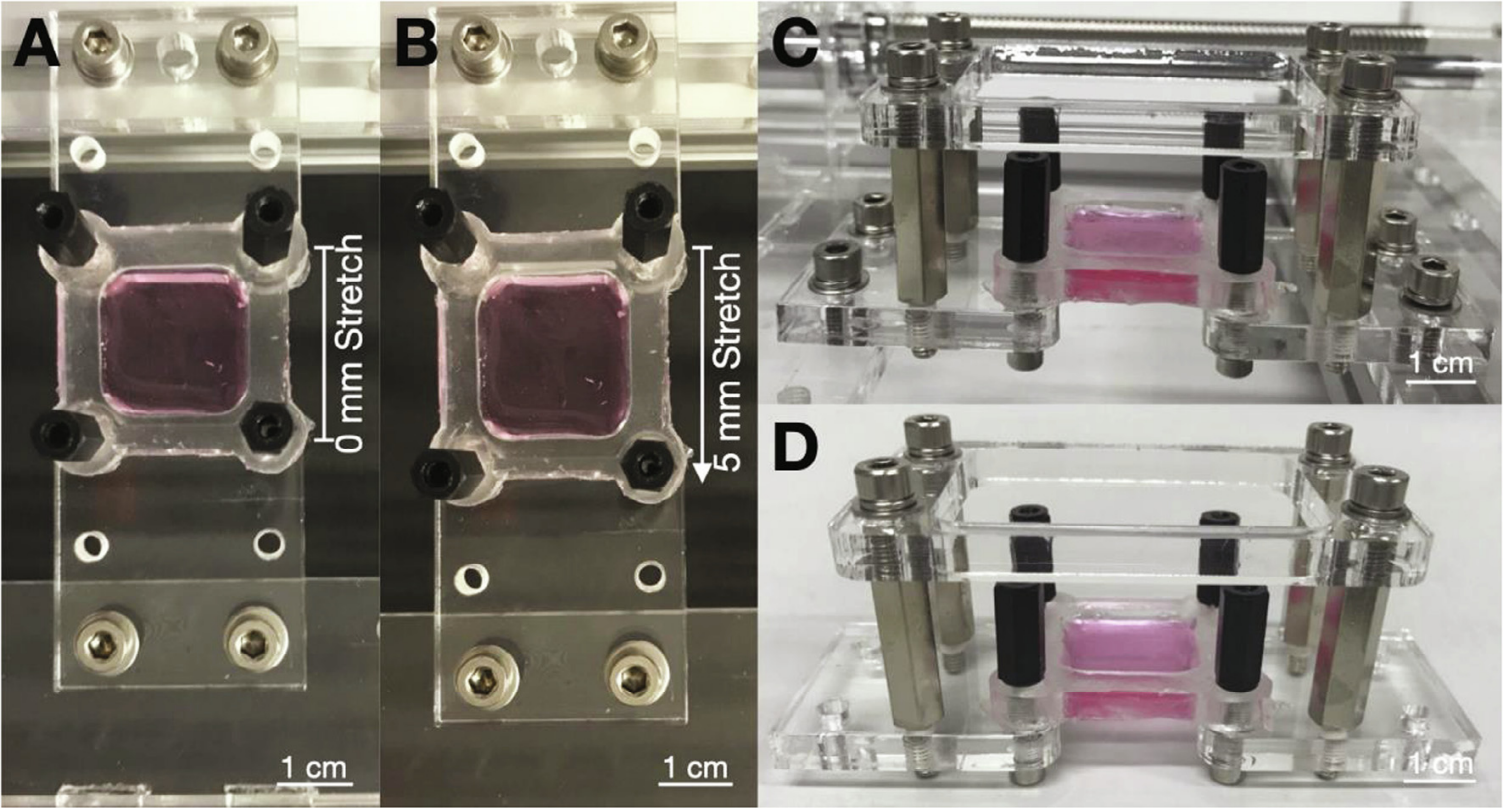
**Stretching of PDMS cell substrates.** A: Cell substrate inserted into the acrylic glass mountings at 0% stretch and fastened with spacer bolts. B: The same sample stretched to 5 mm. C: Acrylic glass frame mounted to the sample with four spacer bolts. D: After unmounting the sample from the stretcher, the acrylic glass frame maintains the 5 mm stretch amplitude.

To perform experiments with the six-well cell stretcher, fabricate PDMS cell substrates and seed cells as explained in Section 6.1. Set the stretcher to zero stretch and attach the PDMS substrate to the stationary and mobile acrylic glass bars with four hooks (Fig. 6A). Secure the PDMS substrates with M3 spacer bolts. However, do not screw the spacer bolts too deeply into the PDMS, otherwise the substrates may rupture easily during stretching. For improved load distribution and increased effective stretch, use 3D printed clamps from the design file *Clamp.stl* to better distribute the mechanical loading across the substrate. This is recommended for very soft substrates and stretching amplitudes higher than 5 mm. Start either one-time stretching or cyclic stretching of the samples using the *pyStretch* software as described in Section 6.2. If the experiment requires further processing or imaging of the samples in a stretched state (Fig. 6B), it is possible to remove the cell substrates from the six-well stretcher while maintaining the stretch. For this purpose, fabricate acrylic glass frames according to the design file *Acryl6MM.pdf*. This file contains one design for narrow frames (for stretch amplitudes of 0–5 mm) and one design for wide frames (for stretch amplitudes of 5–10 mm). To keep the substrate in a stretched state, mount M4 spacer bolts into the central threads of the acrylic glass mounting and attach the acrylic glass frame using M4 screws (Fig. 6C). Unmount the sample from the stretcher; the acrylic glass frames will maintain the stretch (Fig. 6D).

## 7 Validation and characterization

**Fig. 7:**
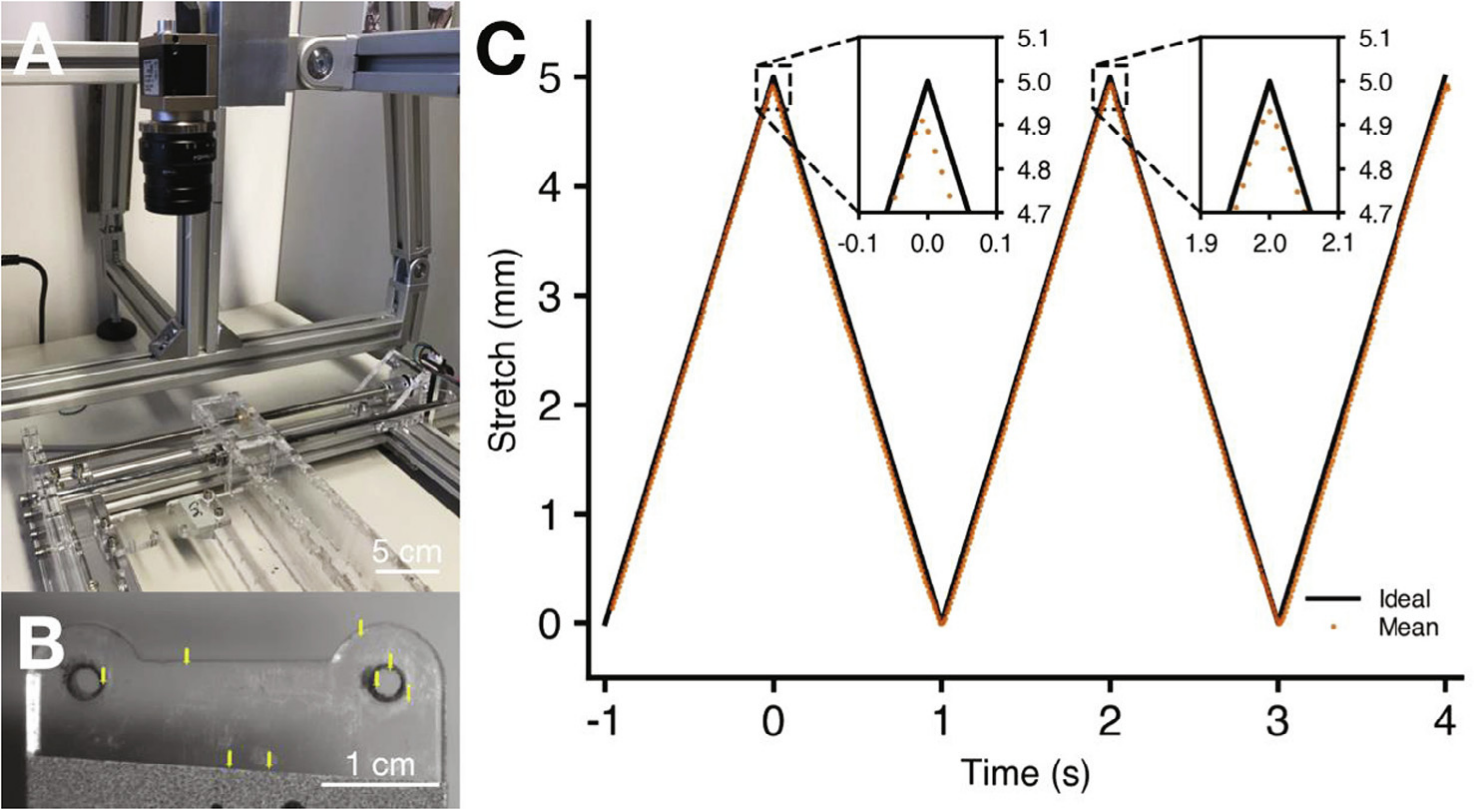
**Verification of cyclic stretch**. A: Setup for imaging the stretcher from top view. B: Still frame of tracked marker points in the video. C: Comparison of evaluated positional data (orange points) with an ideal triangular cyclic stretch (black line). (For interpretation of the references to color in this figure legend, the reader is referred to the web version of this article.)

To validate the cyclic stretching of the six-well cell stretcher, we used a Basler acA4112-30um CMOS sensor camera to image the stretcher movements. We attached an objective with a focal length of 35 mm (Kowa LM35HC) to the camera and mounted it on an aluminum tripod so that one of the moving acrylic glass mountings could be seen in top view (Fig. 7A). We started a cyclic stretch with an amplitude of 5 mm and a frequency of 0.5 Hz and captured 500 images of the moving stretcher with a resolution of 1020 × 196 pixels (corresponding to approx. 60.8 × 11.7 mm) at a frame rate of 100 Hz using the Basler pylon Camera Software Suite. The time of image capture is stored in a time-stamp generated by the camera’s internal clock with 1 ns time resolution. For image analysis, we selected points of high contrast distributed over the entire area of the moving acrylic glass mounting and automatically tracked their movement over the course of the 500 images (Fig. 7B) using the image analysis software ClickPoints [27]. Supplementary Video 2 shows the stretcher movement captured at 100 Hz with the tracked marker points in yellow. We exported the positional data of each tracking point from Clickpoints and evaluated the average vertical displacement (orange data points in Fig. 7C). Compared to the triangular input (target stretch) with an amplitude of 5 mm and a frequency of 0.5 Hz (black line in Fig. 7C), the root mean square error of the output (difference between target and measured stretch) was 91.5 µm (less than 2% relative error) with negligible temporal deviations.

**Fig. 8:**
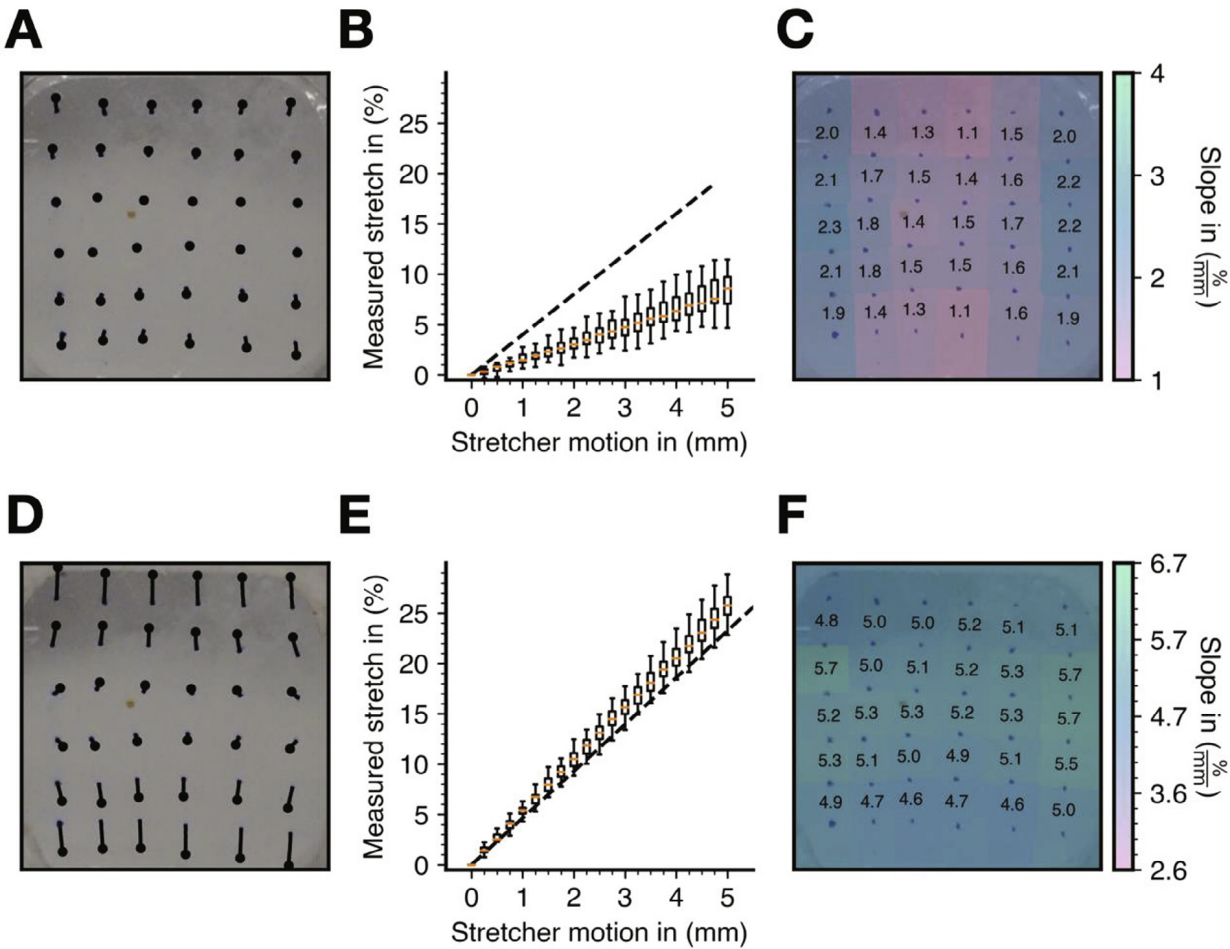
**Uniaxial stretching of PDMS substrates without and with clamps attached**. A: Traces of marker points on a PDMS substrate, which was stretched to a total amplitude of 5 mm in steps of 250 µm without clamps attached. The image shown was recorded at 5 mm stretch. B: Measured stretch of marker points for target stretches between 0 and 5 mm (median (orange line), 25% / 75% percentiles (boxes), 1.5 inter-quantile range (error bars)). Dashed line represents the target stretch assuming a baseline length of 25 mm. C: Spatial distribution of the effective stretch as determined by linear regression from the slope of the local stretch in percent versus mm stretcher motion. (D,E,F): Same as (A,B,C), but with clamps attached to the PDMS substrate for a more even load distribution. The dashed line in (E) is the target stretch assuming a baseline length of 21.5 mm. (For interpretation of the references to color in this figure legend, the reader is referred to the web version of this article.)

Next, we mounted a soft PDMS substrate (1:32 crosslinker-to-base ratio, cured for 6 h, E = 114 kPa) on the cell stretcher and evaluated the transmission of stretch to the substrate. For this, we drew 36 marker points on the surface of the substrate (Fig. 8A), applied uniaxial stretch up to 5 mm in steps of 250 µm and took top-view images with an Olympus OM-D E-M10 camera mounted to a tripod. We evaluated the resulting substrate stretch by tracking the movement of the marker points using the image annotation software Clickpoints (Fig. 8A) and quantified the measured stretch by calculating the relative change in distance between two marker points in stretch direction.

Without the clamps, we found that the effective stretch of all 36 marker points remained substantially below the target stretch of 4%/mm (Fig. 8B). The effective stretch was only (1.68 ± 0.33) %/mm (mean ± STD of n = 30 point pairs) and tended to be larger at the edge of the substrate compared to the center (Fig. 8C). With the clamps in place, the effective stretch was 5.13 ± 0.31%/mm (mean ± STD of n = 30 point pairs) (Fig. 8E), demonstrating that the clamps dramatically improve the transmission of the target stretch to the substrate. We therefore strongly recommend the use of the clamps. But even with the clamps in place, we found that the local stretch tended to be slightly larger at the edge of the substrate compared to the center (Fig. 8F).

As expected for materials with a Poisson’s ratio close to 0.5 (which is the case for PDMS [28]), we saw a noticeable contraction of the substrate perpendicular to the stretch direction (Fig. 8D). In Supplementary Materials S7-11, we verified the reproducibility of the measurement of effective substrate stretch for different setups. In brief, for a given PDMS substrate, the effective stretch was highly reproducible when measured repeatedly at different mounting positions, even after applying one hour of static or cyclic stretch to the substrate. However, there is a noticeable (~9% rms) difference in effective stretch between different PDMS substrates. The average effective stretch for five PDMS substrates with a crosslinker ratio of 1:32 and curing time of 6 h was (4.96 ± 0.46) %/mm (mean ± STD, see Supplementary Material S12). In practical terms, however, this 9% variation in the effective stretch applied to cells in separate samples is unlikely to result in measurable biological responses.

The observation that the measured effective stretch of 4.96 %/mm is larger than the target value of 4.65 %/mm may be caused by the curvature at the corners of the PDMS substrates and the clamps, which slightly reduces the baseline length of 21.5 mm at the corners. Our recommendation is to use the effective stretch value that we calibrated from multiple measurements on different substrates, instead of a theoretical target stretch.

## 8 Maximum stretching frequency

The power and rotational speed of the stepper motors as well as the pitch of the lead screws limit the maximum stretcher speed and acceleration under load. The stepper motors (model number: 42shdc3025-24b) have a maximum angular speed of 1000 rpm without load. Together with the pitch of the Anet A8 lead screws of 8 mm per revolution, this results in a theoretical maximum stretching velocity of 133.33 mm/s. The maximum recommended stretching speed under realistic loading, acceleration and deceleration conditions over prolonged time periods, however, will be considerably lower. As a practical guide, we have tested and verified the proper operation of the stretcher under load for a range of extreme conditions as listed in Table 2. Note that the load was applied for 5 min by manually pressing against the carriage, as stretch amplitudes of 100% would have resulted in the rupture of the elastic substrates.

**Table 2:** Maximum stretching amplitude for different frequencies. The six-well cell stretcher was set to cyclic stretching with the respective combinations of frequency and amplitude, and the number of completed cycles and the time passed were measured independently. Values are given as stretch amplitude (in mm) and the equivalent stretch for PDMS substrates as described in Section 7.

Frequency	Stretch amplitude	Net speed
0.5 Hz	25 mm (116%)	25 mm/s
1 Hz	18 mm (84%)	36 mm/s
2 Hz	6.75 mm (33%)	27 mm/s
3 Hz	3 mm (15%)	18 mm/s
4 Hz	1.75 mm (8.5%)	14 mm/s
5 Hz	1 mm (5%)	10 mm/s

## 9 Example application for cell biological studies

To test the applicability of the six-well cell stretcher for cell biological experiments, we studied the activation of the tension-sensitive transcription factor YAP (Yes-Associated Protein) using two commonly used cell biology techniques: immunofluorescence microscopy and immunoblot analysis. The activation of the YAP transcription factor is known to be regulated by cytoskeletal tension, which can be modulated by biochemical or mechanical cues, such as mechanical stretch, composition of the extracellular matrix, cell contact inhibition, or growth factor stimulation [29], [30], [31], [32], [33], [34]. Here, we combined the mechanical stretch with pharmacological treatment using the myosin inhibitor blebbistatin, and followed the cellular response by YAP monitoring.

We prepared PDMS cell substrates as described in Section 6.1 (1:32 crosslinker ratio, 6 h curing time, E ≅ 114 kPa as measured by indentation, see Section 5.3) and adhesively functionalized the substrates with collagen. Epithelial Madin-Darby Canine Kidney (MDCK) cells were plated on the substrates at a concentration of 250.000 cells/cm^2^ and incubated for 16 h in 1.5 mL of DMEM medium (Sigma-Aldrich, St. Louis, MO) supplemented with 10% fetal bovine serum (Gibco, ThermoFisher Scientific, Waltham, MA) and 1% penicillin/streptomycin (Sigma-Aldrich) in a cell culture incubator (5% CO_2_, 37 °C). Immediately before stretching, cells were either treated with blebbistatin (5 or 20 μM, Sigma-Aldrich) or left untreated. PDMS substrates with cells were then mounted to the cell stretcher as shown in Fig. 6A. PDMS substrates with cell monolayers were stretched with a triangular waveform with 5 mm amplitude at 0.5 Hz for 6 h in a humidified CO_2_ incubator at 37 °C. The PDMS substrates were mounted to the six-well cell stretcher using 3D printed clamps, hence the effective cell stretch was 24.8 ± 2.3% (see Section 7). Afterwards, cells were either fixed with 4% paraformaldehyde (for immunofluorescence analysis) or lysed using RIPA buffer (for Western blot analysis). The RIPA buffer consisted of 20 mM Tris (pH set to 7.4), 150 mM NaCl, 0.1% sodium dodecyl sulfate, 0.5% Na-Deocycholate, 1% Triton X-100, supplemented with protease and phosphatase inhibitors (Roche, Basel, Switzerland).

Samples for immunofluorescence microscopy were then processed using a standard immunofluorescence staining protocol: YAP was immunostained with sc-101199 mouse primary monoclonal antibody (Santa Cruz, Dallas, TX) at 1:100 dilution, and donkey anti-mouse AF-488 secondary antibody (Jackson ImmunoResearch, West Grove, PA) at 1:500 dilution. Nuclei were stained using 2 µg/ml Hoechst dye (bisBenzimide H33258, Sigma-Adrich). Images were captured using a DM6000 microscope (Leica, Wetzlar, Germany) with an APO 100x/NA 1.4 oil objective.

Subcellular localization of YAP was manually analyzed and categorized as either “nuclear” (when YAP was localized predominantly in the nucleus), “cytoplasmic” (when YAP was excluded from nucleus and predominantly localized in the cytoplasm), or “pancellular” (when YAP was homogeneously distributed throughout the cell).

**Fig. 9:**
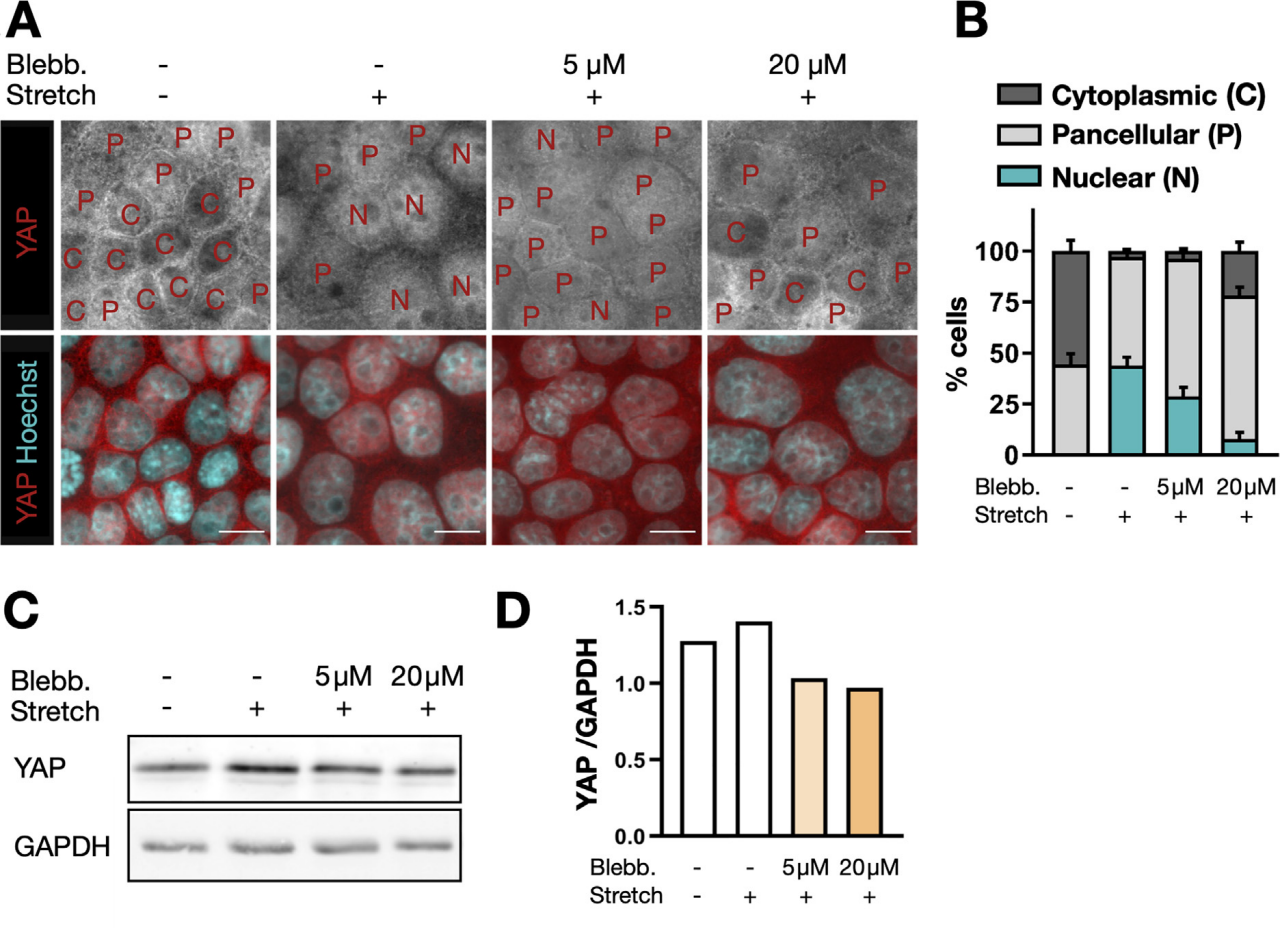
**YAP activation through cyclic uniaxial stretching.** A: Immunofluorescence image of MDCK cells. Cells were either not stretched or cyclically stretched with an effective stretch amplitude of 24.8 ± 2.3% at 0.5 Hz for 6 h, with or without blebbistatin treatment. Letters indicate predominant YAP localization in each cell (C: cytoplasmic, P: pancellular, N: nuclear). Scale bars: 10 μm. B: YAP localization (mean ± SEM from 7 to 10 fields of view per condition) in 451 (control), 506 (stretched, no blebbistatin), 327 (stretched, 5 μM blebbistatin), and 350 (stretched, 20 μM blebbistatin) cells. C: Immunoblot analysis of YAP expression levels in MDCK cells treated as in A and B. D: YAP expression levels normalized to GAPDH expression.

In agreement with previous reports [35], [36], we found that mechanical stretching leads to the activation and nuclear translocation of the tension-sensitive transcription factor YAP in nearly 50% of the cells (Fig. 9A,B). By contrast, in non-stretched cells, YAP was localized mostly in the cytoplasm or throughout the entire cell. The effect of stretch-dependent YAP activation was partially blocked by myosin inhibition using blebbistatin (Fig. 9A,B).

The coefficient of variation in local YAP expression (cytoplasmic fraction) across different field-of-views was 26% for unstretched samples and increased to up to 104% during stretch (Fig. 9). However, this large variability results from random fluctuations between the analyzed cells (~50) in each field-of-view, as demonstrated by the observation that the measured variability agrees with the values predicted from a Bernoulli distribution. The large local fluctuations in YAP distribution can therefore not be attributable to the small local variations in absolute stretch magnitude across a clamped substrate, which varied by only ± 2.3% for a 24.8% stretch.

As mechanical stretch is known to lead to increased YAP expression [33], [37], [38], we monitored the expression level of YAP using immunoblot analysis. This was done with an sc-101199 mouse primary monoclonal antibody (Santa Cruz) at 1:1000 dilution and a donkey anti-mouse IRDye 680RD secondary antibody (LI-COR, Lincoln, NE) at 1:10.000 dilution. For normalization, the housekeeping protein GAPDH was monitored using a G9545 rabbit polyclonal primary antibody (Sigma-Aldrich) at 1:20.000 and a donkey anti-rabbit IRDye 800CW secondary antibody (LI-COR) at 1:10.000 dilution. The Western blot signal was imaged using an Odyssey imaging system (LI-COR). Consistent with the results from the immunofluorescence analysis, we observed increased YAP expression upon stretch, which was blocked in the presence of blebbistatin (Fig. 9C,D).

## 10 Conclusion

We designed an open source six-well cell stretcher, which can be built from parts of an *Anet A8* 3D printer for less than 400€. The stretcher imposes uniaxial stretch with arbitrary waveform and frequency to cells seeded on flexible substrates fabricated from PDMS, and is operated using fully customizable open-source software. We have operated the six-well stretcher on a nearly daily basis (>1h of operation per day) over more than one year and experienced no technical problems on major components such as motors and moving parts that are exposed to high humidity conditions. However, we noticed that the acrylic holders for attaching the flexible substrates developed small cracks around the metal screws. Although thus far they have remained mechanically intact, over longer time periods and for heavy-duty use they may better be replaced with parts made from more durable materials (such as PVC or aluminum).

We demonstrate the applicability of our device as a tool to study mechano-biological processes by investigating the activation of the YAP mechanotransduction pathway in response to cyclic stretch. The possibility of stretching up to six samples simultaneously allows users to combine mechanical stimulation with different drug treatments, biological conditions, and modes of analysis including fluorescence microscopy and immunoblotting.

## Declaration of Competing Interest

The authors declare that they have no known competing financial interests or personal relationships that could have appeared to influence the work reported in this paper.
