# Supplementary material for: A low-cost uniaxial cell stretcher for six parallel wells
Source: HardwareX. 2020 Dec 9;9:e00162. doi: 10.1016/j.ohx.2020.e00162 (PMC9041267; doi:10.1016/j.ohx.2020.e00162)
Supplement: Supplementary data 1 [file mmc1.docx]

**SUPPLEMENTAL DATA S1-5**

**Title:** A low-cost uniaxial cell stretcher for six parallel wells

**Authors:** Delf Kah^1,*^, Alexander Winterl^1^, Magdalena Přechová^2^, Ulrike Schöler^3,4^, Werner Schneider^1^, Oliver Friedrich^3,4^, Martin Gregor^2^, and Ben Fabry^1^

**Affiliations:** ^1^Biophysics Group, Department of Physics, Friedrich-Alexander University Erlangen-Nürnberg (FAU), Erlangen, Germany, ^2^Laboratory of Integrative Biology, Institute of Molecular Genetics of the Czech Academy of Sciences, Prague, Czech Republic, ^3^Institute of Medical Biotechnology, Department of Chemical and Biological Engineering, FAU, Erlangen, Germany, ^4^School in Advanced Optical Technologies, FAU, Erlangen, Germany, *Corresponding author.

**Contact email:** delf.kah@fau.de

The following is a detailed description on how to build and operate the six-well cell stretcher. The required tools (apart from standard tools such as wrenches, Allen keys, etc.) and materials are listed at the beginning of each section. We recommend that all screws for acrylic glass parts are gently tightened by hand and not with an electric screwdriver.

**Contents**

- **S1 Preparations**
- **S2 Stretcher unit**
- **S3 Electronics and controller unit**
- **S4 Molds and fabrication of PDMS substrates**
- **S5 Operation instructions**

# **S1 Preparations**

## **S1.1 Check Anet A8 parts**

Make sure that all components shown in Fig. S1A are included in your *Anet A8* set. With a screwdriver, carefully pry the Z axis nut supports apart (Fig. S1B) and push out the guide rod bearings (Fig. S1C). Use an M2.5 Allen key to loosen the lead screw nuts (Fig. S1C). For calibrating the zero-stretch position, choose either the X or Y limit switch, as their cables are longer than that of the Z limit switch.


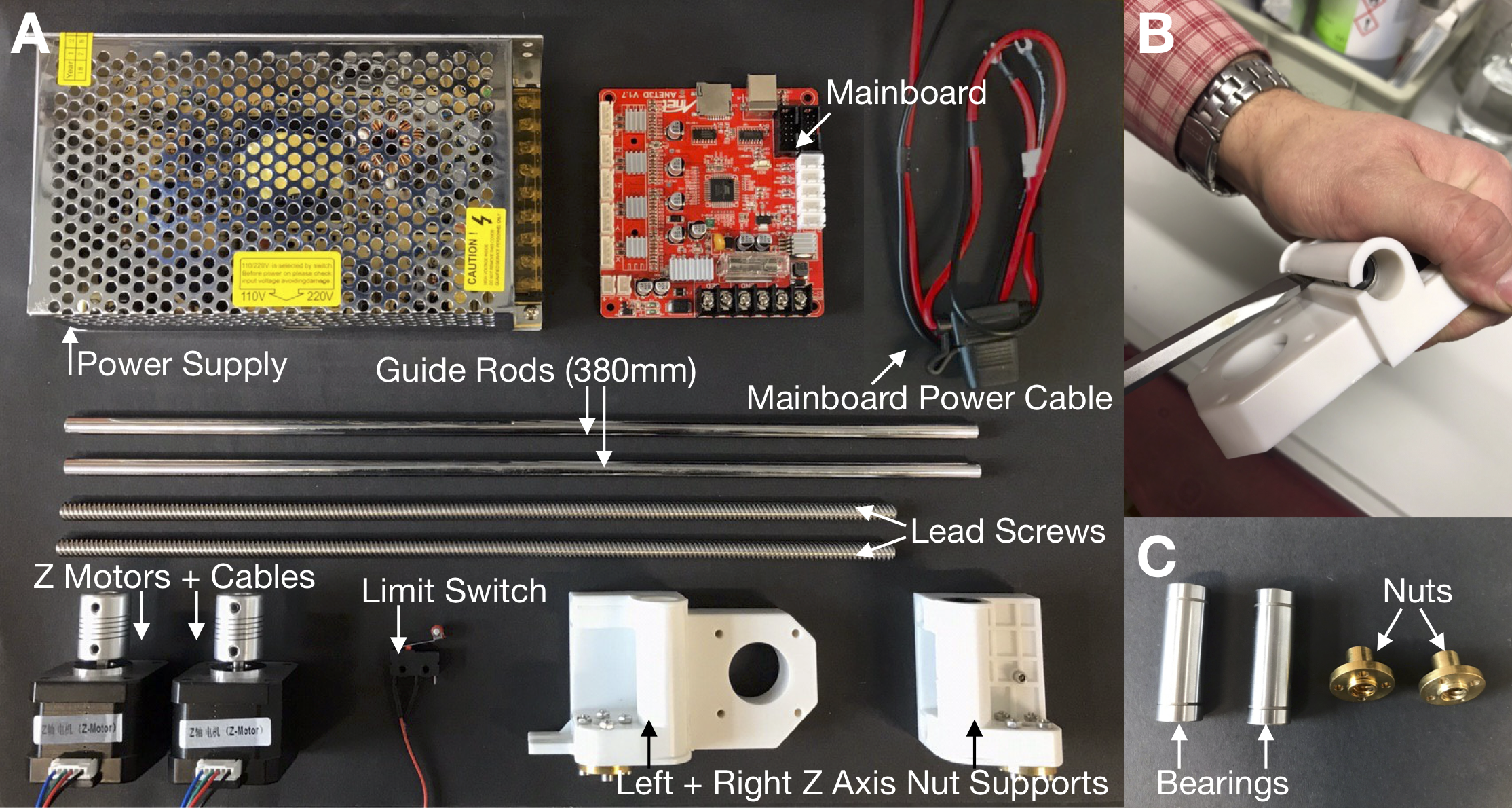


**Fig. S1: Parts needed from the *Anet A8* kit.**

## **S1.2 Prepare acrylic glass parts**

**Tools:**

- **Laser cutting machine**
- **Metric plug taps (M2.5, M3, M4, M5)**

**Material:**

- **3x acrylic glass sheets 600mm x 300mm x 3mm**
- **3x acrylic glass sheets 600mm x 300mm x 6mm**

We provide two design files called *Stretcher3MM* and *Stretcher6MM* as .pdf and .svg files. These are compatible with most programs for laser cutters to produce the acrylic parts for the cell stretcher from material of 3 mm or 6 mm thickness. Three 600 mm x 300 mm sheets of acrylic glass are needed for each thickness. After cutting, manually remove any material stuck at edges and in holes. Check that you have all parts shown in Fig. S2 (6 mm material) and Fig. S3 (3 mm material). Cut threads into the acrylic glass parts as indicated in the legends of Fig. S2 and S3. In the following, individual acrylic glass parts are referred to as labelled in Fig. S2 and S3.


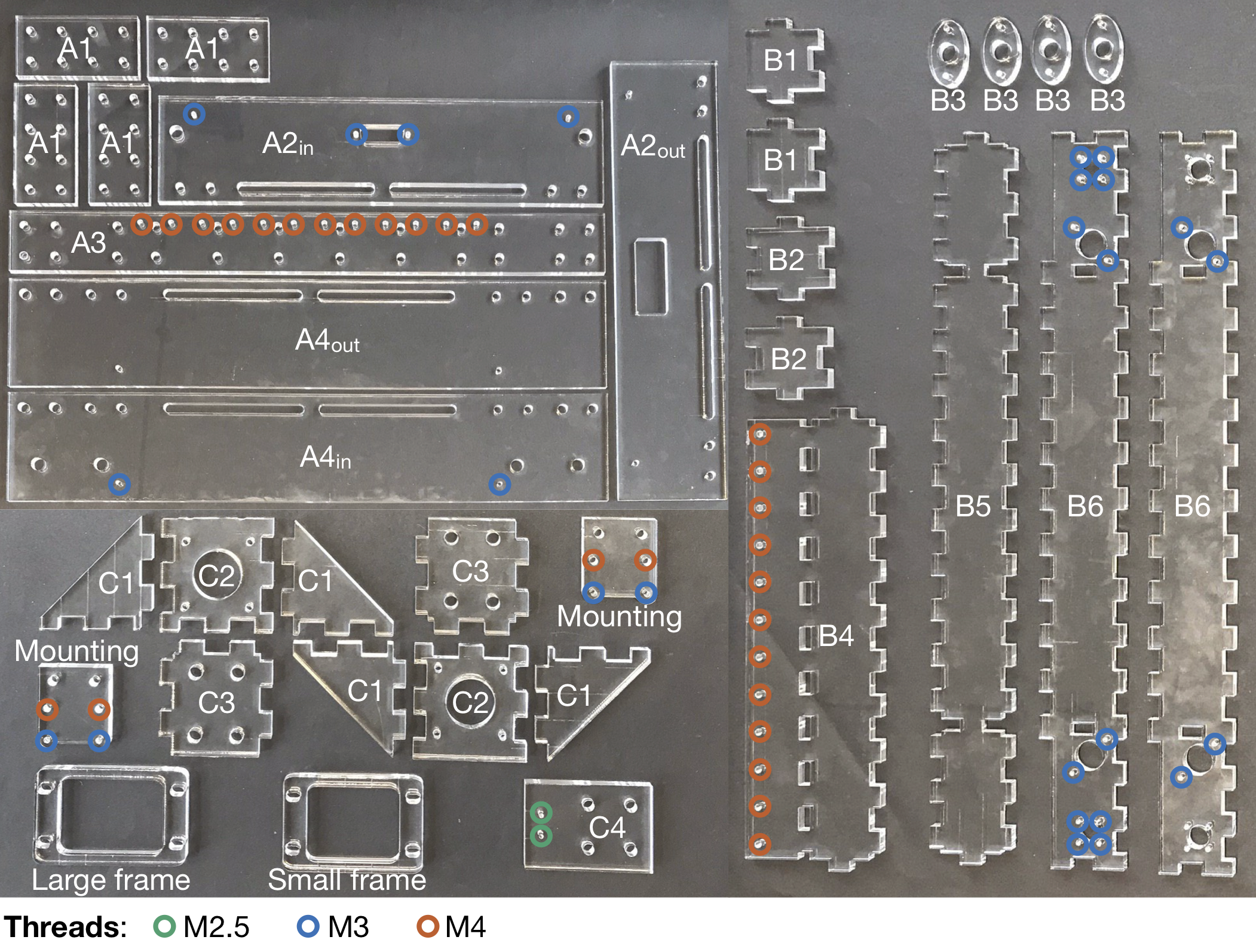


**Fig. S2: Acrylic glass parts (6 mm).** Colored circles indicate the thread size. A total of twelve mountings are required to stretch six PDMS substrates simultaneously (shown here are only two mounting sets). The design file contains six large and six small frames (shown here is only one per size).


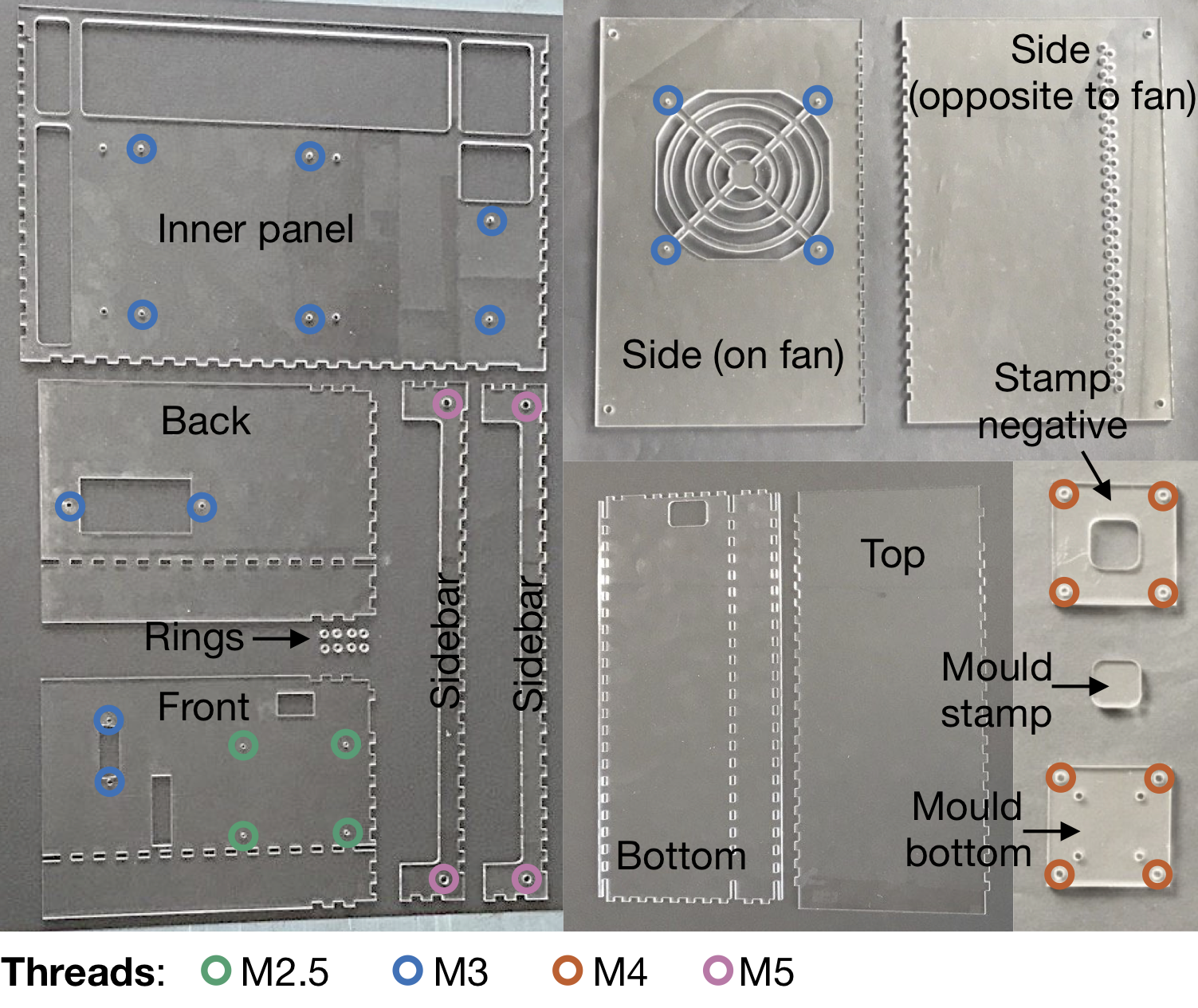


**Fig. S3: Acrylic glass parts (3 mm).** Colored circles indicate the thread size. Several mold parts (bottom and stamp) can be produced to cast multiple PDMS substrates simultaneously.

## **S1.3 Raspberry Pi setup**

We provide a custom version of the open-source *Raspbian* operating system (version: Raspbian Buster 2020-02-13) for the Raspberry Pi computer with *PyStretch*, the software to operate the six-well cell stretcher, with all necessary Python packages pre-installed. For the installation, follow these steps:

- Download the operating system file *Stretcher.zip* (http://dx.doi.org/10.17632/mgkbmytx63.1), as well as the file *Raspberry_Pi_Imager_v1.2.zip* (http://dx.doi.org/10.17632/mgkbmytx63.1) to a Windows PC.
- Extract *Raspberry_Pi_Imager_v1.2.zip*, run the installation file *Raspberry_Pi_Imager_v1.2/imager.exe* and follow the installation instructions. Then, run the newly installed application *Raspberry Pi Imager* [1].
- Insert a microSD card of at least 8 GB into your computer. In the Raspberry Pi Imager application (Fig. S4), select the option “*ERASE”* from the menu *Operating System* and select the system path of your microSD card in the menu *SD Card*. Press *WRITE* to format your microSD card.
- Next, select the option *“Use custom”* from the menu Operating System and select the system path the file “Stretcher.zip”. Also, select the system path of your microSD card in the menu *SD Card*. Press *WRITE* to install the operating system to the microSD card. This takes about 30 minutes.


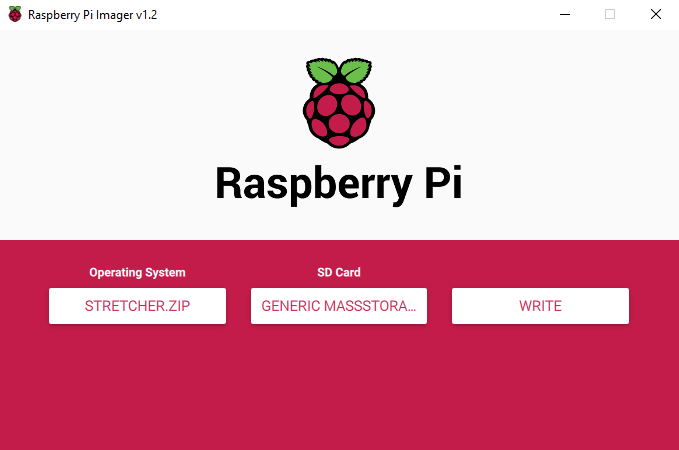


**Fig. S4: Graphical user interface of *Raspberry Pi Imager*.**

## **S1.4 Anet firmware setup**

It is necessary to replace the firmware of the Anet A8 mainboard in order to be functional as a cell stretcher. Specifically, we need to change the default step size of the z-Motors in order for them to be fast enough for our purposes. To do this, follow these steps:

- Connect the Anet A8 mainboard to a Windows PC via USB connection (in Fig. S14C this is illustrated for a connection to a Raspberry Pi).
- Open the Windows Device Manager. If the Anet A8 mainboard is listed as *“USB-Serial CH340”*, remember the corresponding communication port (COM6 in Fig. S5A) and skip the next step. If the Anet A8 mainboard is recognized as *“USB2.0-Serial”* (Fig. S5B) or *“Unknown Device”*, follow the next step to install the correct driver.
- The Anet A8 mainboard is based on the popular CH340 chip for microcontrollers. The driver for the CH340 chip can be found on the website of the Chinese manufacturer (http://www.wch.cn/download/CH341SER_EXE.html, cited 2020 14/05/2020) or re-distributed from several other sources on the internet. Download and run the executable driver file and follow the installation instructions.
- Download the files Skynet_for_Stretcher.zip (http://dx.doi.org/10.17632/mgkbmytx63.1) and Arduino-1.8.0.zip (http://dx.doi.org/10.17632/mgkbmytx63.1) and extract both files.
- Open the Arduino application [2] by running the executable file *Arduino-1.8.0/arduino.exe*.
- In the Arduino application select File>Open. Select and open the file *Skynet_for_Stretcher/SkynetV2.3.1/SkynetV2.3.1.ino* [3].
- Under Tools>Board select “Anet V1.0” (Fig. S6A).
- Under Tools>Port select the Anet A8 manboard’s communication port (Fig. S6B).
- Transfer the modified firmware to the Net A8 mainboard by clicking on the right pointing arrow in the upper left corner (Fig. S6C).


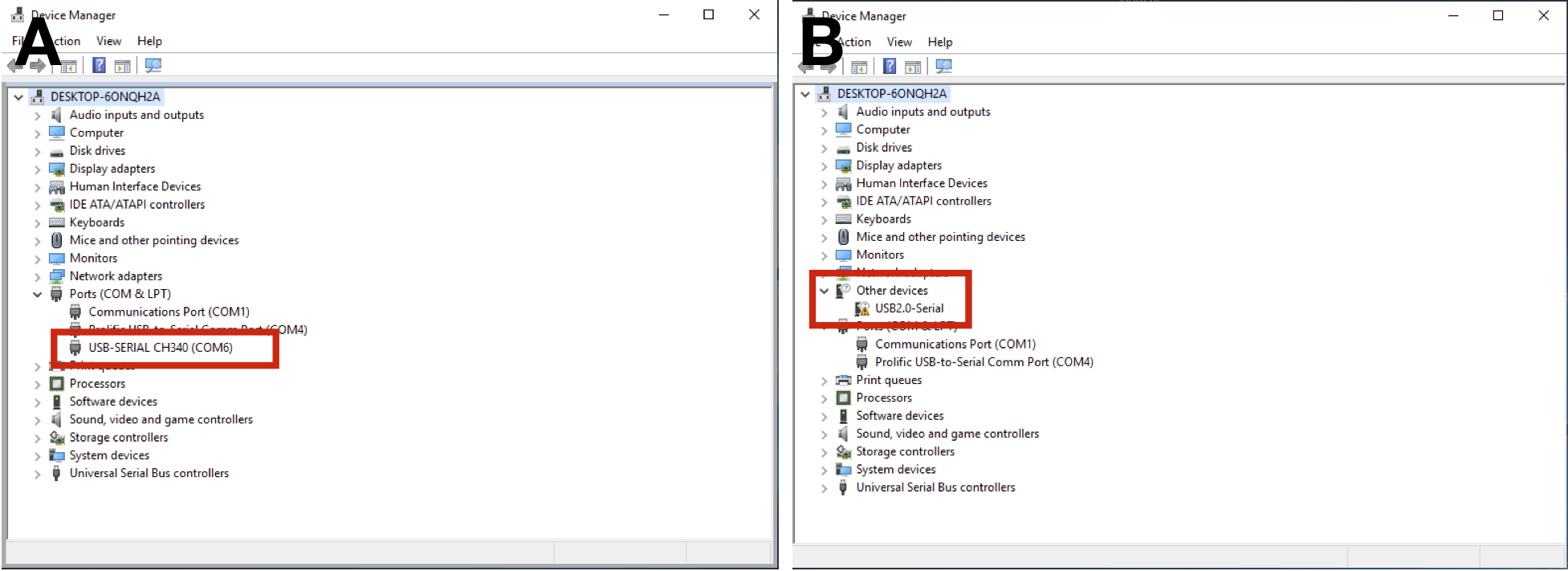


**Fig. S5: Recognizing the Anet A8 mainboard in the Windows Device Manager.**

**
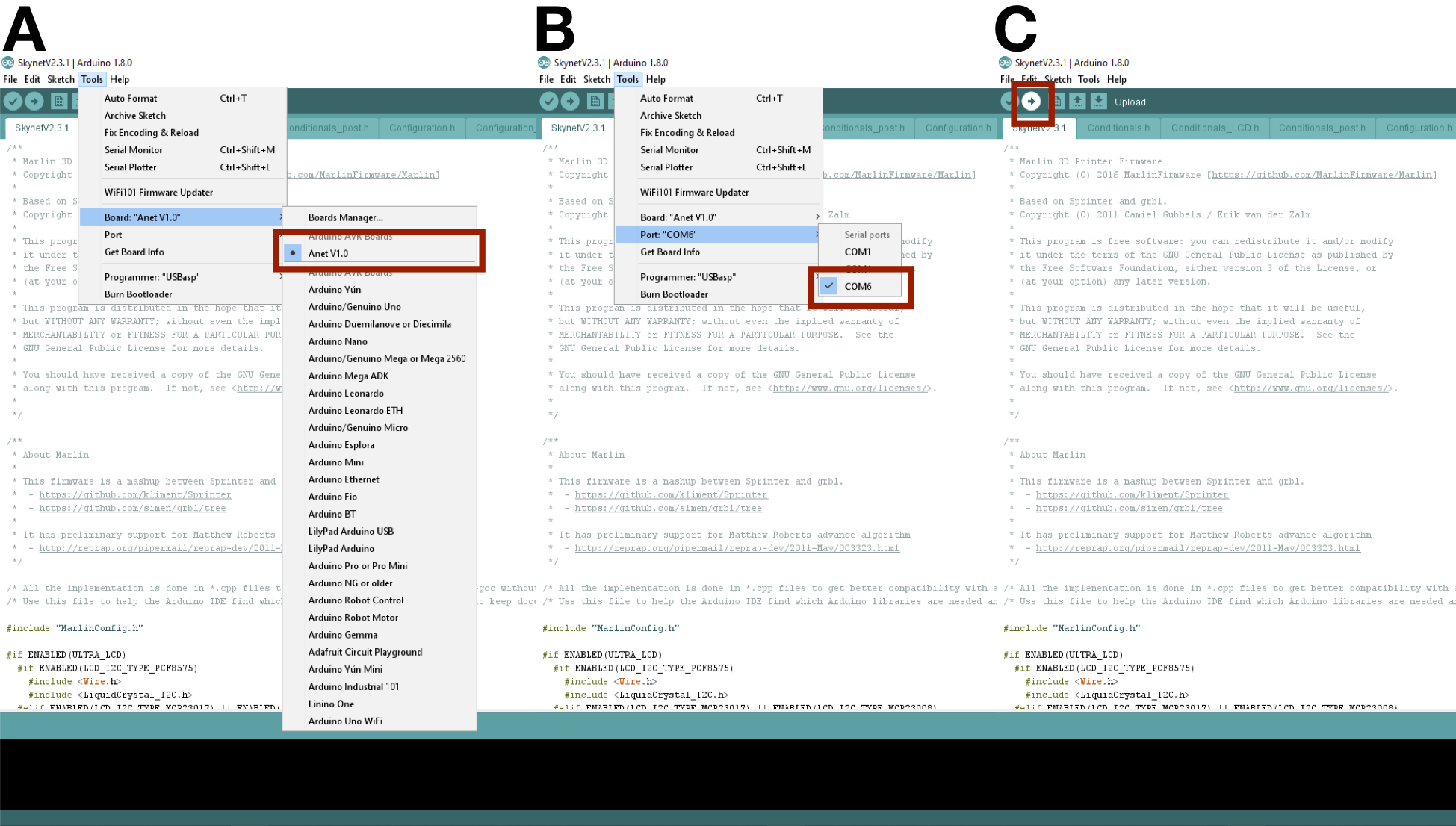
**

**Fig. S6: Installing the Anet A8 firmware with the Arduino integrated development environment.**

# **S2 Stretcher unit**

## **S2.1 Mobile bar**

**Tools:**

- **UV light source**

**Material:**

- **Acrylic glass parts: B1 (2x), B2 (2x), B3(4x), B4, B5, B6 (2x)**
- **Anet A8 Parts: Bearings (2x), Nuts (2x)**
- **Acrylic adhesive**
- **Screws: 4x (M3x10mm + spring washers), 4x (M3x12mm + spring washers + washers)**

**Optional, but useful: Toothpicks, plastic bowl for adhesive, precision angle for 90 degree alignment of the glued parts, anti-corrosion oil**

The mobile bar is the carriage of the uniaxial stepper motor. It is glued together from the acrylic glass parts B1-B6 with *Acrifix 192* adhesive, which has to cure under UV light for 20 minutes. Acrylic adhesive must be applied to all contact surfaces between the parts to be joined. For this purpose, it is practical to first pour the adhesive into a small plastic bowl and then apply it to the parts with a toothpick. Note that the adhesive hardens quickly when exposed to air, even without curing by UV light. In our experience, it is difficult to glue together the entire mobile bar in one go. Especially the first walls have to be aligned carefully and cured quickly after gluing.

We suggest to carry out the gluing and curing in several steps as illustrated in Fig. S7:

- First glue the inner walls (2x B2) to the top part (B5) of the mobile bar (Fig.4A,B). By plugging in (but not gluing!) one of the side walls (B6) you can align the glued parts by 90 degrees. Align the top and side wall (preferably with a suitable precision angle) at 90 degrees (Fig. S7C) and cure the parts for about 10 minutes under UV light. It is okay if the angles shield some of the glued areas, as these areas will later be exposed to UV light again. At this step, it is only important that enough of the adhesive areas cure so that the inner walls harden at a 90 degree angle with respect to the top part.
- Next, remove the side wall (B6), which you plugged in before, from the other parts. Put adhesive on all contact surfaces of the top part (B5) and inner walls (2x B2) on one side where they meet a side wall (Fig. S7D). Carefully insert the sidewall (Fig. S7E), fix the top and sidewall at a 90 degree angle and cure the adhesive under UV light as before.
- As in the previous step, apply adhesive to the surfaces where the top part (B5) and inner walls (2xB2) have contact to the missing sidewall (B6) (Fig. S7F). You can also apply the adhesive to the individual side wall (Fig. S7G). Carefully join the sidewall to the rest of the mobile bar.
- The previously cured adhesive areas should be sufficient to hold the remaining parts of the mobile bar at 90 degree angles. Therefore, you can now glue the remaining three parts in one go: Two end pieces (2x B1) and the bottom part (B4) (Fig. S7H). Apply adhesive to the contact surfaces of each part with the rest of the mobile bar and press the parts together firmly. If the parts do not hold tightly enough, cure them while fixing them with suitable angles or clamps. Note that the bottom part (B4) is not symmetrical and extends to one side of the mobile bar. This extending side must be opposite the sidewall (B6) with the M3 threads for the lead screw bearings (see Fig. S2). If you have mixed up the side parts, you can also carefully drill threads into the finished mobile bar afterwards. Cure the mobile bar one last time under UV light for 20 minutes (Fig. S7I).
- Mount nut holders (2x B3) at each end of a sidewall (B6) with a total of four M3x12mm screws, spring washers, and washers. Lubricate both nuts from the *Anet A8* kit with anti-corrosion oil and insert them into the mobile bar at the open ends (Fig. S7J). Then screw the two remaining nut holders with a total of four M3x12mm screws, spring washers, and washers (see position in Fig. S7K).
- Mount one bearing from the *Anet A8* kit at each end of a side panel (B6) with a total of four M3x10mm screws and spring washers (see position in Fig. S7K). Standard M3 washers are too large to be used here. The bearings must be mounted on the side wall opposite the extending bottom of the mobile bar (see the red outlines in Fig. S7L).


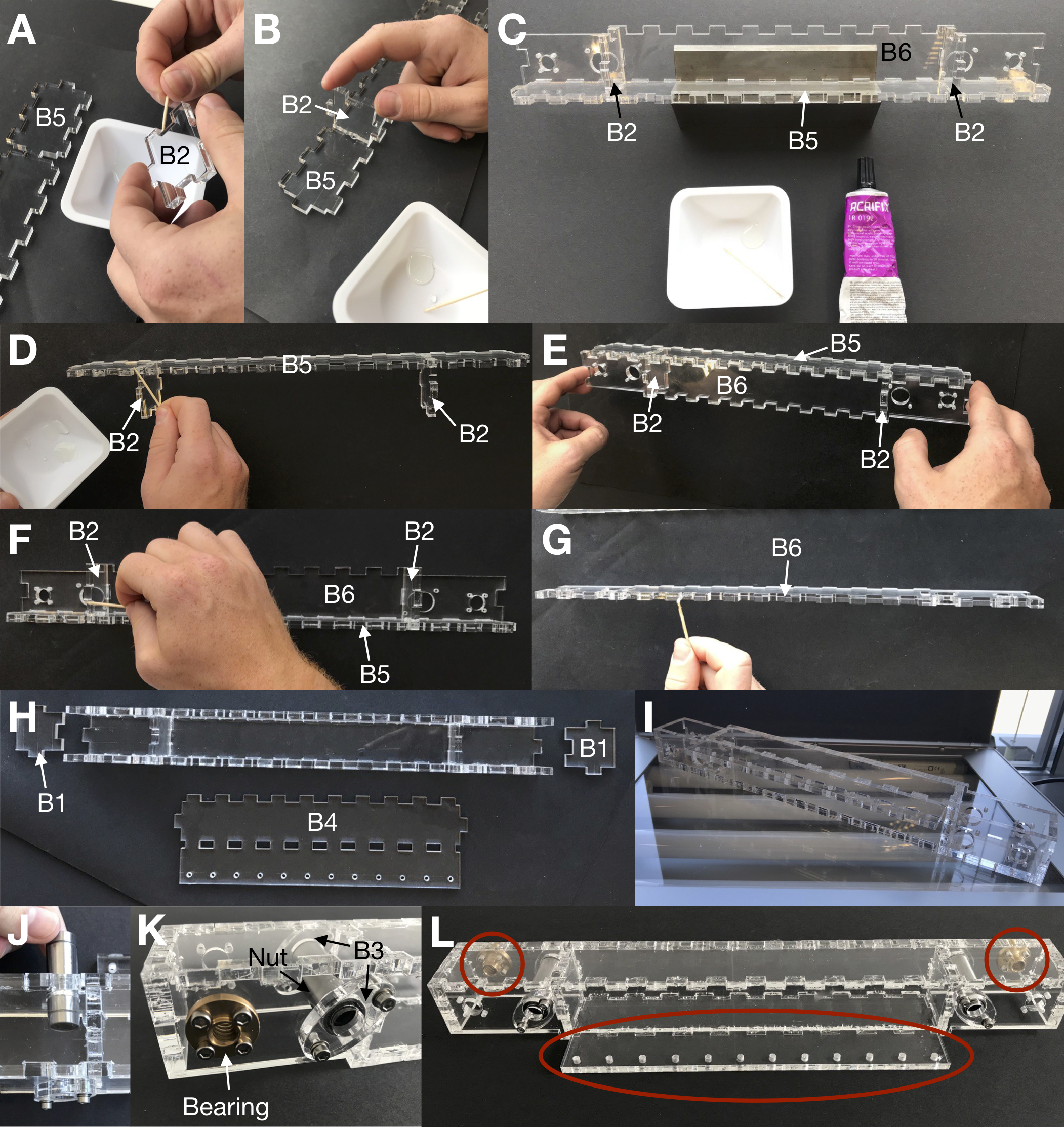


**Fig. S7: Assembly of the mobile bar.**

## **S2.2 Frame: Part I**

**Tools:**

- **Metric plug tap (M6)**

**Material:**

- **Acrylic glass parts: A1 (2x), A3, A4_in_, A4_out_**
- **Aluminum profile 440 mm (2x), aluminum profile 310 mm (1x)**
- **Slim M5 T-slot nuts (30x)**
- **Screws: 2x (M3x12mm + spring washers + washers), 24x (M5x12mm + spring washers + washers), 4x (M5x20mm + spring washers + washers), 4x (M6x20mm + spring washers + washers)**
- **2x (M5 rubber feet + spring washers + washers)**

In this section, we describe how to assemble the stationary end of the stretcher frame (stationary bar). Here and in the following, acrylic parts and aluminum profiles are often connected by T-slot nuts and M5 screws. Note that T-slot nuts are available in two different designs: a wider nut that can only be inserted into the aluminum profiles from the end, and a slimmer nut that can be inserted into the profiles at any position. Here, we use the slim T-slot nuts and insert them in profiles that are already screwed together at the sides. If you use the wider T-slot nuts, they need to be inserted before the aluminum frames are screwed together.

- Cut two M6 threads (at least 7 mm deep) into each side of both long aluminum profiles (440 mm) (Fig. S8A).
- Insert a total of eight T-slot nuts into the short aluminum profile (310 mm) (see positions in Fig. S8B). Then screw together two acrylic parts (2x A1) in parallel alignment with the aluminum profile (Fig. S8C). Use a total of eight M5x12mm screws, spring washers, and washers, which you only screw on loosely so that the acrylic glass parts can still be moved over the aluminum profile.
- Insert four T-slot nuts in each long aluminum profile (at the end where you cut the threads).
- Connect all three aluminum profiles to the acrylic parts from the previous steps with six additional M5x12 screws and two M5 rubber feet (each attached with spring washers and washers) so that the aluminum profiles are aligned in a U-shape (Fig. S8D). Tighten all screws, making sure that the aluminum profiles are connected at a 90° angle.
- Connect the acrylic glass parts A4_in_ and A4_out_ with two M3x12mm screws, spring washers, and washers (Fig. S8E).
- Insert four T-slot nuts into the outer side of the short aluminum profile (310 mm). Connect the acrylic glass parts A4_in_+A4_out_ with four M5x20mm screws and four M6x20mm screws (each with spring washers and washers) to the stretcher frame (Fig. S8F). The acrylic part A4_out_ must be on the outside of the U-shape and the acrylic part A4_in_ on the inside.
- Finally, attach the acrylic glass part A3 as shown in Fig. S8G. Use a total of ten T-slot nuts, M5x12mm screws, spring washers, and washers. Before tightening the screws, make sure that the acrylic parts A3 and A4_in_ are flush with each other. The threads of the acrylic part A3 are exposed to particular mechanical stress during operation of the cell stretcher and, according to our experience, can break over time if the corresponding screws are overtightened. So be especially careful here. You can alternatively use washers made of Teflon or PVC.


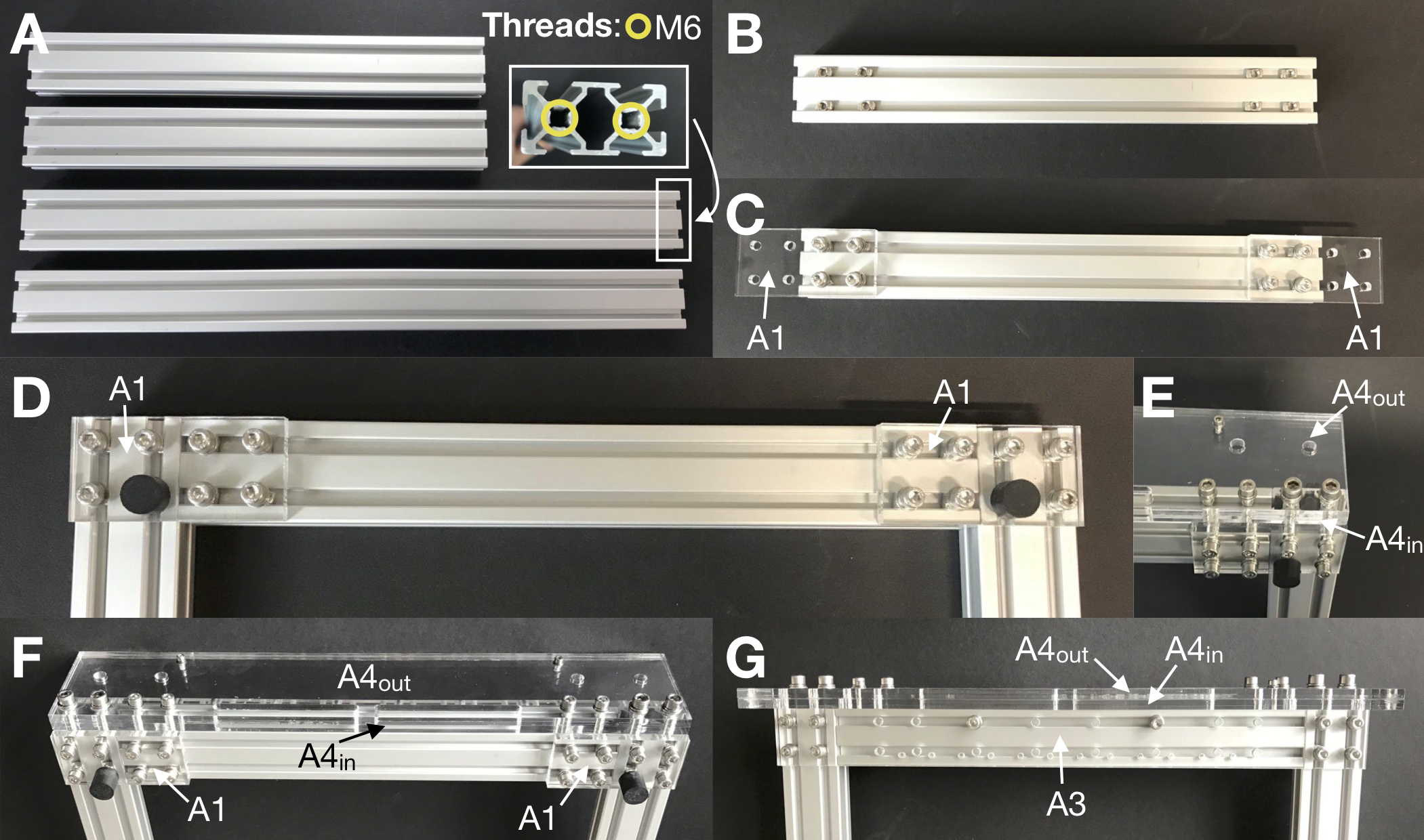


**Fig. S8: Assembly of the stationary bar of the stretcher frame.**

## **S2.3 Frame: Part II**

**Material:**

- **Acrylic glass parts: A1 (2x), A2_in_, A2_out_, mobile bar**
- **Anet A8 Parts: guide rods (2x)**
- **Aluminum profile 310mm (1x)**
- **Slim M5 T-slot nuts (20x)**
- **Screws: 2x (M3x12mm + spring washers + washers), 14x (M5x12mm + spring washers + washers), 4x (M5x20mm + spring washers + washers)**
- **2x (M5 rubber feet + spring washers + washers)**
- **D-SUB connector (male) + screw set (2x)**

**Optional, but useful: (Rubber) hammer, an assistant**

In this section, we describe how to assemble the second end of the stretcher frame and how to mount the mobile bar and guide rods in between.

- Connect the acrylic glass parts A2_in_ and A2_out_ with two M3x12mm screws, spring washers, and washers.
- Mount the D-SUB connector (male) to the acrylic glass part A2_in_+A2_out_ with two sets of screws (see Fig. S9A for orientation).
- Insert four T-slot nuts into the left short aluminum profile (310mm) (Fig. S9A). Use four M5x20mm screws, spring washers, and washers, which you only screw on loosely so that the acrylic glass parts can still be moved over the aluminum profile.
- Screw together the remaining two acrylic parts (2x A1) in parallel alignment with the aluminum profile (Fig. S9A). Use eight T-slot nuts, as well as a total of eight M5x12mm screws, spring washers, and washers, which you only screw on loosely so that the acrylic glass parts can still be moved over the aluminum profile.
- Place the stretcher frame on its side so that the open end is accessible. Insert eight T-slot nuts into the open ends of each of the long aluminum profiles and connect the acrylic/aluminum part from the last steps to the rest of the stretcher frame (Fig. S9B). Use a total of six M5x12mm screws and two M5 rubber feet, each of which is loosely fixed with spring washers and washers, so that the unit can still be moved within the aluminum profiles. The D-SUB connector must face outwards.
- Insert the guide rods from the *Anet A8* kit through the bearings of the acrylic glass mobile bar. Hang the mobile bar in the stretcher frame by inserting the ends of the guide rods into the holes provided in the inside of the acrylic glass end pieces. The extending end of the mobile bar must be facing the stationary end (the bottom of the U-shape) of the stretcher frame (see Fig. S9 C&D).
- Place the stretcher upright, firmly press down the moveable end and tighten all screws and rubber feet (Fig. S9C).
- Centrally align the movable acrylic end piece as precisely as possible within the short aluminum profile (Fig. S9D). Ideally, the mobile bar and the fixed end of the stretcher frame should be flush over the entire length (highlighted in red in Fig. S9D). Small changes in position can be made by carefully tapping with a (rubber) hammer. Then fix the four screws that connect the acrylic end part to the aluminum profile.


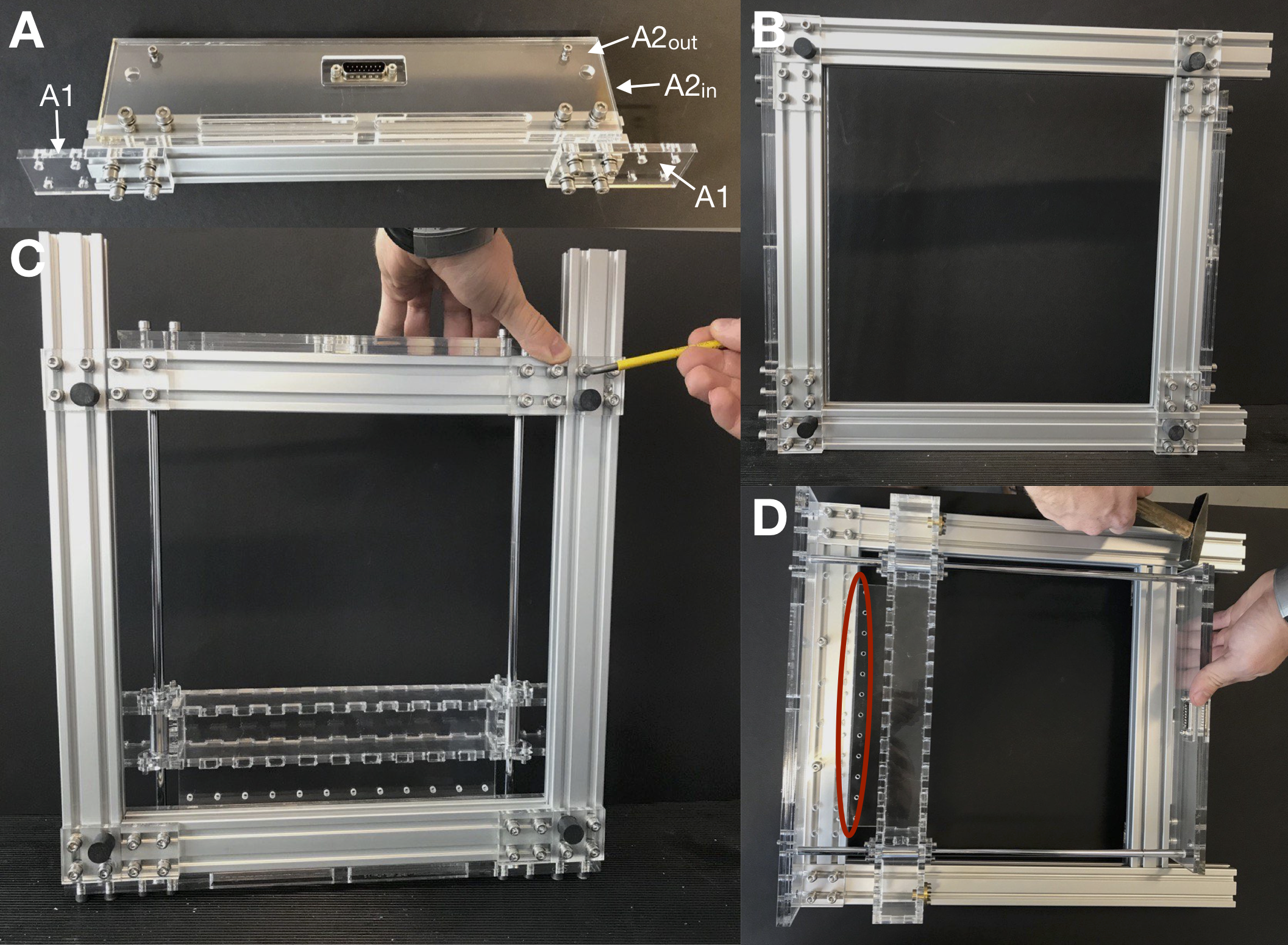


**Fig. S9: Assembly of the second end of the stretcher frame and mounting of the mobile bar.**

## **S2.4 Motors**

**Tools:**

- **UV light source**
- **Narrow slotted screwdriver**

**Material:**

- **Acrylic glass parts: C1 (4x), C2 (2x), C3 (2x), C4**
- **Anet A8 Parts: Z Motor (2x), lead screws (2x), limit switch**
- **Acrylic adhesive**
- **Slim M5 T-slot nuts (12x)**
- **Screws: 2x (M2.5x10mm), 8x (M3x12mm + spring washers + washers), 12x (M5x12mm + spring washers + washers)**

This section describes how to assemble the last parts of the stretcher unit by adding the two stepper motors and the lead screws.

- Assemble an acrylic mounting frame for each of the two stepper motors from parts C1 (2x), C2 and C3 (Fig. S10A). Proceed as in Section S2.1 and use acrylic adhesive, which is cured under UV light.
- Attach each stepper motor from the *Anet A8* kit with four M3x12mm screws, spring washers, and washers to a mounting frame. Note the cable orientation in Fig. S10B.
- Stick four M4x12mm screws each (with spring washers and washers) through the holes at the bottom of the acrylic motor mountings and loosely screw four T-slot nuts at the bottom (Fig. S10B). The motor mounting frames must not be inserted into an aluminum profile at this point.
- Loosen the grub screws on both motors as shown in Figure 7C until the lead screws can be inserted.
- Turn the two lead screws from the *Anet A8* kit through the nuts of the mobile bar by hand until they are completely inserted into the holes provided for them on the acrylic end piece (Fig. S10D).
- Slide the two motors in their acrylic mounting frames into the aluminum profiles so that the motors pick up the free ends of the lead screws (Fig. S10E).
- Press the motors on the lead screws while tightening the grub screws and M5 screws (Fig. S10F).
- Take the limit switch from the *Anet A8* kit and remove the protruding metal lever. To do this, take a narrow slotted screwdriver and slide the small tab on the limit switch casing as shown in Fig. S10G, opening the housing. Remove the metal lever (Fig. S10H) and click the limit switch housing back together. Alternatively, you can simply bend the metal lever with force until it breaks, as it is no longer needed.
- Fix the limit switch to the acrylic glass part C4 using two M2.5x10mm screws. The red button must face towards the mobile bar.
- Centrally attach the acrylic glass part C4 with the limit switch on the short aluminum profile underneath the D-SUB connector as shown in Fig. S10I. Use four M5x12mm screws, spring washers, washers, and T-slot nuts.


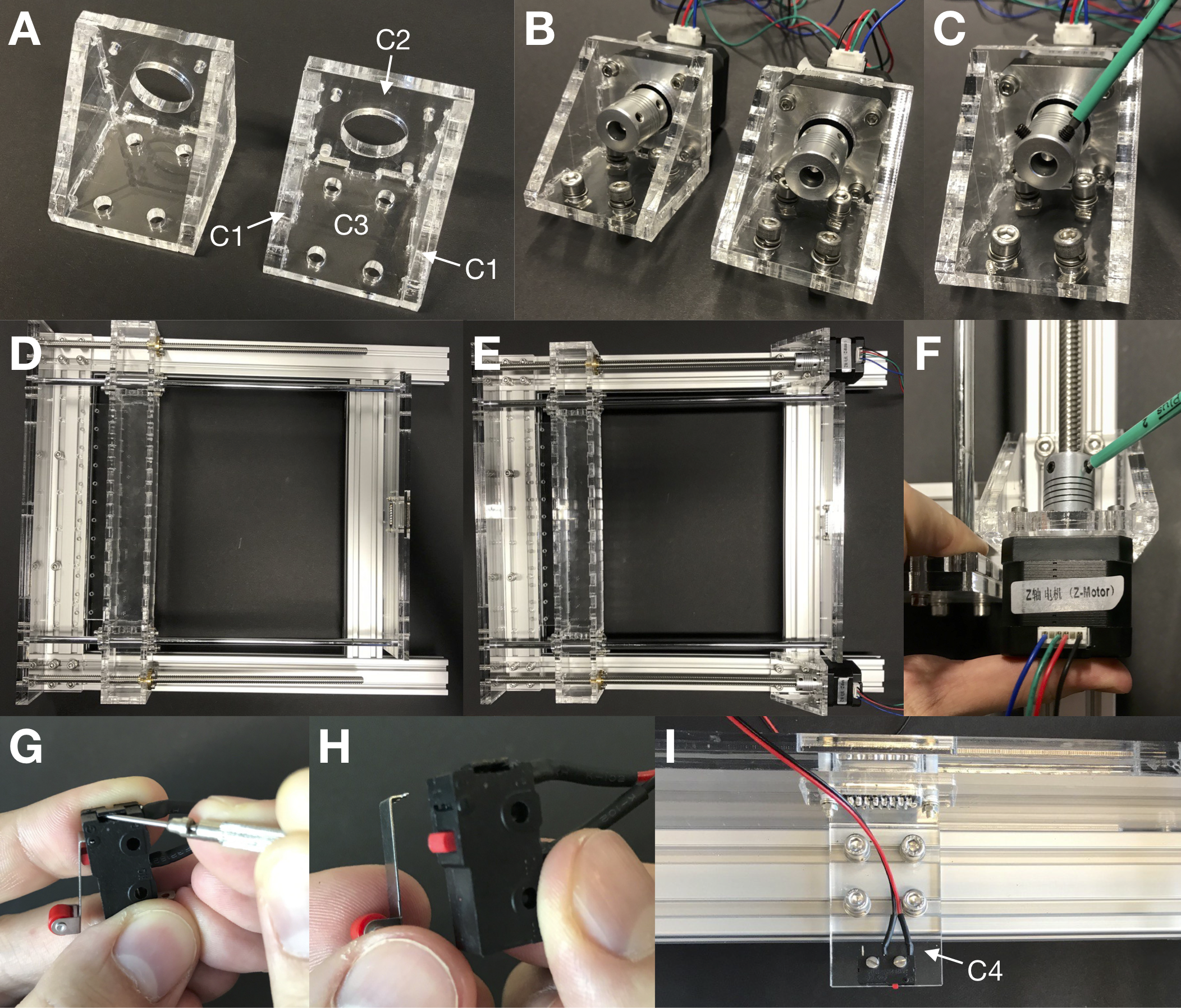


**Fig. S10: Assembly of the stepper motors, the lead screws, and the limit switch.**

## **S2.5 Wiring**

**Tools:**

- **Scissors**
- **Wire stripping tool**
- **Soldering iron**

**Material:**

- **Solder**
- **Tape**
- **D-SUB connector (female)**

In this section, we describe how the two stepper motors and the limit switch are wired and soldered to the D-SUB connector.

- Cut the stepper motor cables to a length of about 40 cm. Cut the cable of the limit switch to a length of about 10 cm. Strip the ends of all cables over a length of approx. 3 mm (Fig. S11A).
- Solder all ten cables (2x4 stepper motor cables + 2 limit switch cables) to the D-SUB connector (Fig. S11B). The D-SUB connector is already installed and is held in position by the stretcher unit, which makes soldering easier. A suggestion for the cable positions is shown in Fig. S11C.
- Attach the cables to the acrylic glass frame with tape (Fig. S11D). Form small cable loops in front of the soldering points for strain relief, as well as behind the stepper motors, in case that you want to readjust them later.
- Plug in the second D-Sub connector (female). At this point, the cables that have just been cut off can be soldered for later use. Strip the ends of all cables over a length of approx. 3 mm. Make sure that you solder cables of the same color from the same motor (or limit switch) to opposite pins (Fig. S11E).


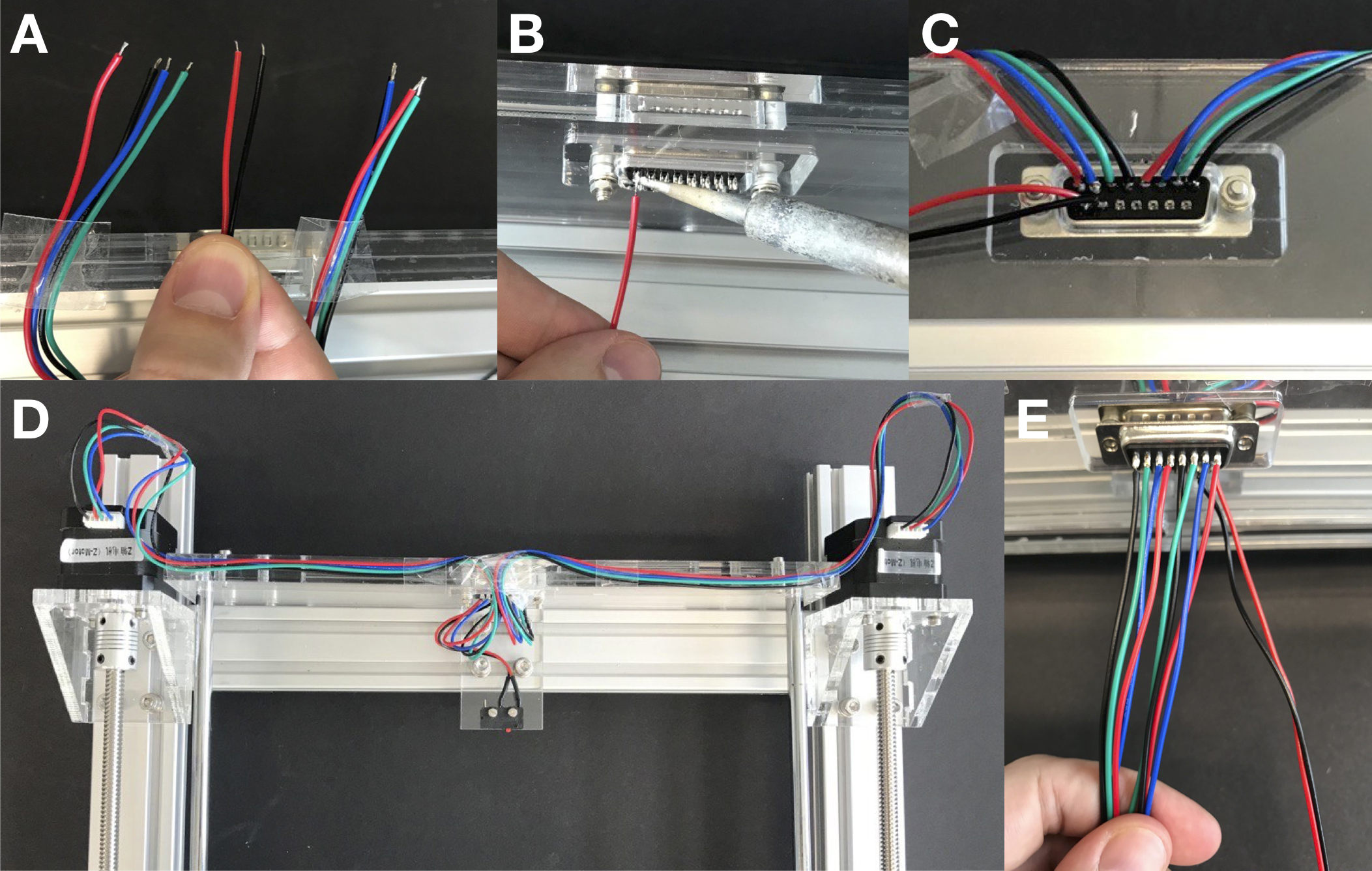


**Fig. S11: Wiring of the stretcher unit.**

# **S3 Electronics and controller unit**

## **S3.1 Housing**

**Tools:**

- **UV light source**

**Material:**

- **Acrylic glass parts (3mm): Bottom, inner panel, sidebar (2x), back, front, top, side (opposite to fan), side (on fan)**
- **Acrylic adhesive**

**Optional, but useful: M5x10mm screws (4x), file, straight weights to align the acrylic glass**

In this section, we describe how to assemble the housing of the electronics and controller unit. To do this, acrylic glass parts must be glued together and cured under UV light as explained in previous sections. Compare the parts closely with the illustrations, as it is possible to glue parts together mirror-inverted. Again, we recommend to glue and cure the parts in several steps.

- Glue the inner panel and the two sidewalls to the bottom. Pay attention to the asymmetry of the inner panel and the additional holes in the sidewall, which should be placed on the side opposite to the fan (compare the color coded holes in the inner panel and the position of the sidewalls in Fig. S12A).
- Next, add the front and back panels. Pay particular attention to the correct alignment of the parts (compare the color coded holes in the inner panel in Fig. S12B).
- Finally, glue together the cover consisting of three parts. It is practical to screw the two sides (fan and opposite to fan) to the corresponding sidewalls with a total of four M5x10mm screws. Note the asymmetry of the side panel with ventilation slots for the fan (compare with Fig. S12C&D). It may not be possible to bring the sides into the correct position, because cured adhesive residues from the previous curing steps stick out. In this case, carefully remove the cured adhesive with a file.
- Glue the top part to the sides while it is resting on the rest of the case. It is practical to hold the sides in position with long, straight weights when curing (Fig. S12C).
- The finished acrylic glass housing with cover is shown in Fig. S12D.


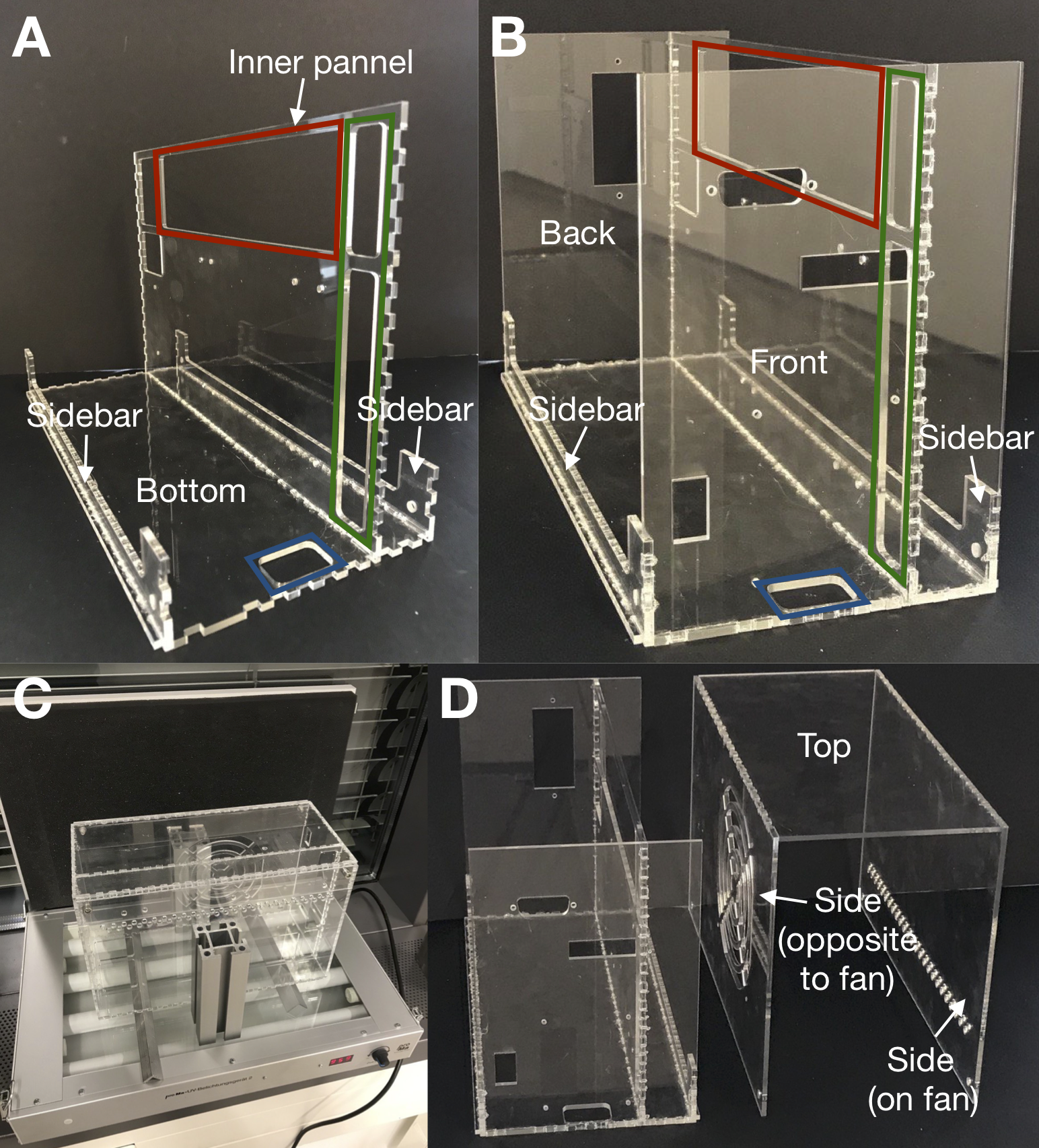


**Fig. S12: Assembly of the electronics and controller unit case.**

## **S3.2 Installing components**

**Material:**

- **Acrylic glass parts: Rings (8x)**
- **Anet A8 Parts: Power supply, mainboard**
- **Raspberry Pi 4B**
- **USB angle adapter DOWN (2x)**
- **HDMI angle adapter**
- **Micro SD card**
- **DC/DC converter**
- **Filtered power entry module**
- **2A micro fuse (2x)**
- **D-SUB connector (female) + screw set (2x)**
- **Fan and anti-vibration pads**
- **Screws: 4x (M2.5x6mm + spring washers + washers), 8x (M3x8mm + spring washers + washers), additional 8x M3x8mm**

**Optional, but useful: 3 pin header, red and black conductor cables (ca. 10 cm each), heat-shrink tubing, ferrules (and suitable pliers)**

In this section, we describe how to install the electronic components for the electronics and controller unit into the acrylic glass housing.

- Plug two USB connectors, one HDMI connector, and the SD card into the Raspberry Pi (Fig. S13A). Connect the Raspberry Pi with four M2.5x6mm screws, spring washers, and washers with the acrylic glass housing (Fig. S13B). Place four of the 3mm acrylic glass rings as spacers between the Raspberry Pi and the acrylic glass housing.
- Mount the power supply with four M3x8mm screws, spring washers, and washers to the acrylic glass housing. See Fig. S13C for the orientation of the power supply.
- Mount the mainboard with four M3x8mm screws, spring washers, and washers to the acrylic glass housing (Fig. S13D). Place four of the 3mm acrylic glass rings as spacers between the mainboard and the acrylic glass housing.
- Mount the mainboard with four M3x8mm screws, spring washers, and washers to the acrylic glass housing (Fig. S13D).
- Mount the DC/DC converter with two M3x8mm screws to the acrylic glass housing (Fig. S13D).
- Insert two 2A micro fuses into the power entry module. Then connect it to the acrylic glass housing with two M3x8mm screws (Fig. S13E).
- Mount the female DSUB connector, to which you have soldered the cables in Section S2.5, to the acrylic glass housing with two screw sets (Fig. S13F).
- Mount the fan with four anti-vibration pads and four M3x8mm screws on the inside of the cover (Fig. S13G). Orient the fan so that it sucks air into the case.
- Prepare the fan cable for the next section. We recommend building an adapter that allows the fan to be plugged in and out while the end of the cable is firmly connected to the power supply (Fig. S13H). Ideally, such an adapter consists of a pin header that can be plugged into the 3 pin socket at the end of the fan cable. Alternatively, you can also cut the fan cable, strip the 3 wires, and build an adapter from a screw terminal. We recommend to use ferrules on the end of the adapter, but it is also possible to simply stick the cable strands into the power supply. Note that the fan has a wire for voltage (red) and grounding (black) as well as a wire for regulating the rotation speed (yellow). We do not need this third wire and it is best to insulate this cable.


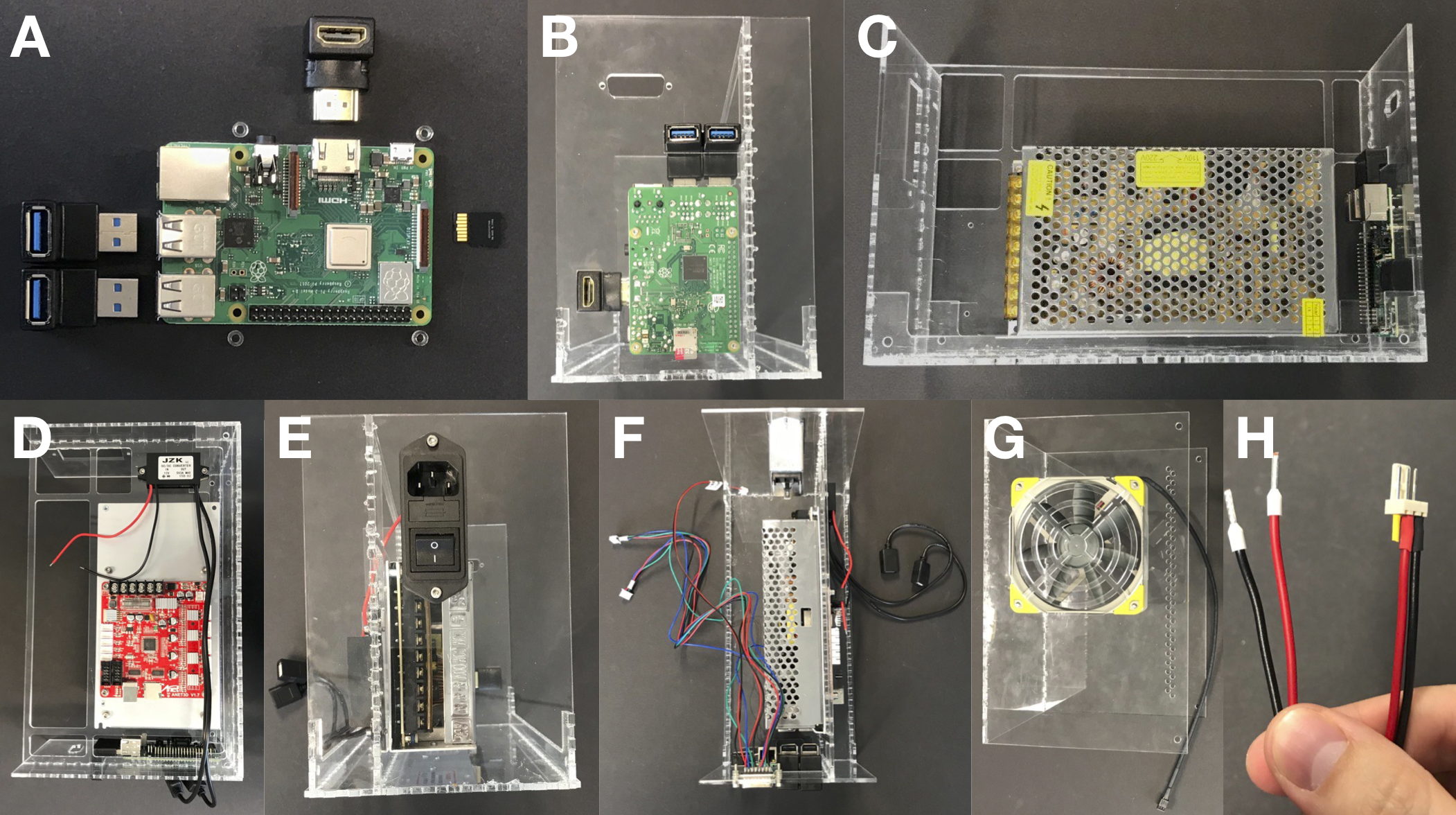


**Fig. S13: Installing components into the electronics and controller unit housing.**

## **S3.3 Wiring**

**Material:**

- **Anet A8 Parts: Mainboard power cable**
- **Blue, brown, and green/yellow conductor cables (ca. 20 cm each)**
- **6.3mm/15mm^2^ cable lugs (3x) and suitable crimping tool**
- **Ferrules (3x)**
- **Micro USB to USB (Type A, male) angled Adapter (DOWN, at least 30cm)**
- **Screws: 4x M5x10mm**

**Optional, but useful: Wire stripper, USB (Type A, male) to USB (Type B, male) cable (30cm)**

In this section we describe how the electrical components of the electronics and controller unit are wired together.

- Prepare connections from the power entry module to the power supply unit (Fig. S14A). Use approx. 20 cm long conductive cables, preferably following the standard color scheme: blue for neutral (N), brown for single phase line (L), and green/yellow striped for ground (PE). Strip all cable ends. Crimp a cable lug to one end of each cable and a ferrule to the other end (you can also just use the cable strands). Use the cables to connect the power entry module to the power supply according to Fig. S14B. The N and L lines are interchangeable, but the PE line must be in the correct position for both components.
- Connect the mainboard to the power supply with the mainboard power cable from the *Anet A8* kit. Screw the red cable (+) at the power supply into one of the +V slots and the black cable (-) into one of the COM slots. Follow Fig. S14C for the wiring on the mainboard.
- Connect the open cable ends of the DC/DC converter to the power supply using the same color scheme as in the previous step. Connect the other end of the DC/DC converter to the micro USB port of the Raspberry Pi with an angled USB connector.
- Connect the fan to the power supply using the cable adapter from the previous section (connect the red cable to +V and the black cable to COM).
- Connect the mainboard (USB Type B port, see Fig. S14C) to the Raspberry Pi (USB Type A port). The *Anet A8* kit comes with a cable suitable for this purpose.
- Connect the remaining ends of the cables previously soldered to the DSUB connector to the mainboard (Fig 11C). Make sure that both the limit switch and the motors are connected to the respective Z-axis slots.
- Make sure that the fan is connected to the power supply, put on the housing cover, and mount it to the rest of the electronics and controller unit with four M5x10mm screws.


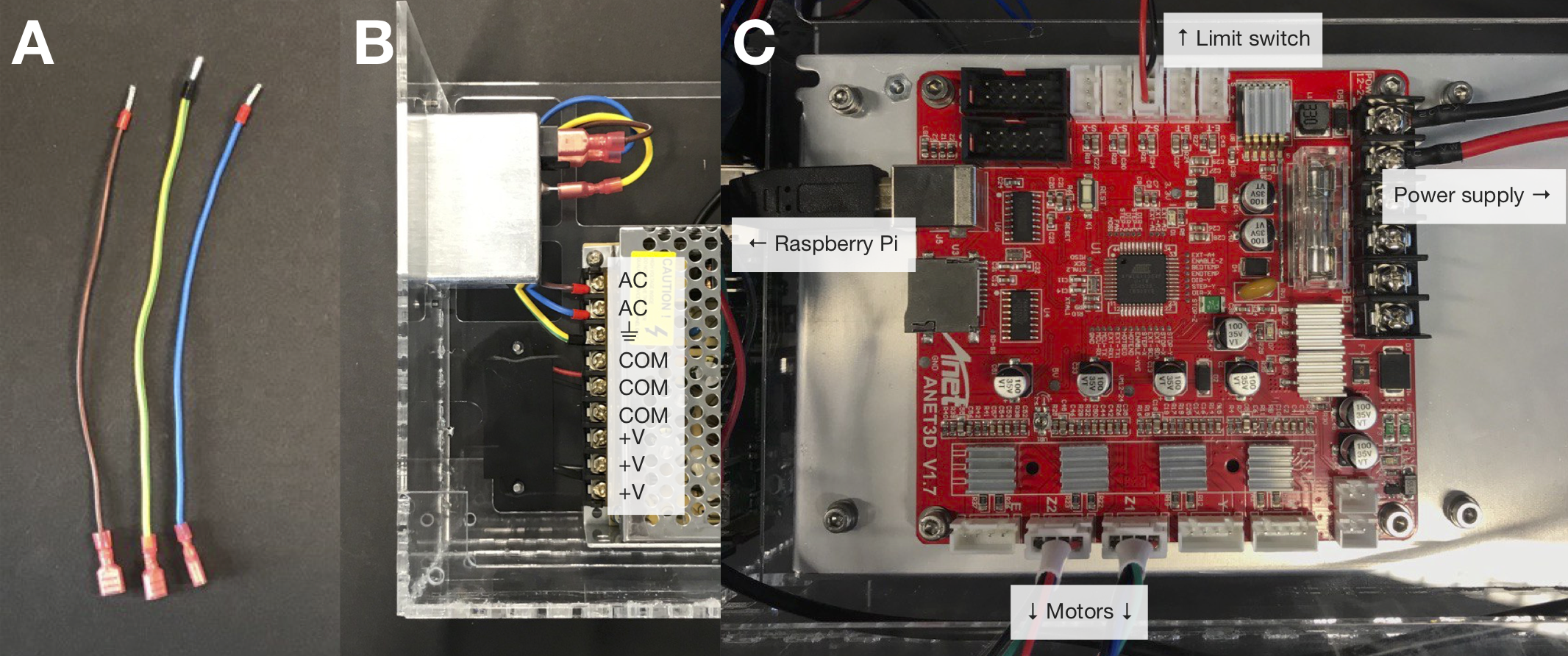


**Fig. S14: Wiring of the controller unit.**

# **S4 Molds and fabrication of PDMS substrates**

**Tools:**

- **UV light source**
- **Scissors**
- **Oven**
- **Scalpel**

**Material:**

- **Acrylic glass parts: mold bottom, mold stamp, stamp negative**
- **3D printed mold frame**
- **Acrylic adhesive**
- **Plastic straws (3 mm diameter)**
- **Screws: 4x (M4x10mm + spring washers + washers + nuts)**
- **SYLGARD™ 184 Silicone Elastomer Kit (Dow Chemical)**
- **Plastic container and stirring tool**

**Optional, but useful: Trichloro(1H,1H,2H,2H-perfluorooctyl)silane (Sigma)**

In this section, we describe how to assemble a mold for the fabrication of cell substrates out of polydimethylsiloxan (PDMS). Since manufacturing of a single PDMS substrate involves curing for 24 hours in an oven, it is useful to make multiple molds for parallel fabrication of multiple substrates. The finished mold consists of two parts: a stamp (+bottom) and a frame. The thickness of the stamp determines the height of the chamber, in which cells are eventually seeded. If the stamp is made of 3 mm thick acrylic glass as described here, the chamber for cell seeding holds just over 1.5 mL of culture medium, which is sufficient for most biological applications. The capacity can be increased by a thicker stamp. The thickness of the mold frame in turn determines the thickness of the bottom of the PDMS substrate. Due to the high refractive index of PDMS (1.41), a thick substrate bottom can be a problem when imaging with inverted microscopes. To achieve the thinnest possible substrate bottom, we recommend the production of the mold frame by 3D printing, for which we provide a design file in the .stl format for printing a frame with a thickness of 3.5mm, resulting in a bottom thickness of 0.5mm. The frame thickness in the 3D print design file can easily be adjusted.

- Screw the acrylic mold bottom and the acrylic stamp negative together with four M4x10mm screws and nuts (Fig. S15A). Carefully apply a film of acrylic adhesive to one side of the acrylic glass mold stamp and press it carefully into the screwed mold negative. Allow the adhesive to cure under UV light for 20 minutes and remove the stamp negative (Fig. S15B).
- Screw the glued acrylic stamp and 3D printed mold frame together with four M4x10mm screws, spring washers, and washers (Fig. S15C). Cut a plastic straw with 3 mm diameter into four pieces of about 2 cm length. Insert the straw pieces into the four inner holes of the substrate mold.
- If you find that the PDMS substrates are difficult to remove from the mold after curing, the mold can be coated before use. One possibility is to evacuate a desiccator to approx. 10 mbar with the mold inside and to pipette 10µl trichloro(1H,1H,2H,2H-perfluorooctyl)silane into it (Fig. S15D).
- Fabricate PDMS with the SYLGARD™ 184 kit by mixing curing agent and base in a plastic container (Fig. S15E). The curing agent to base ratio determines the stiffness of the cured PDMS substrate. With a mixing ratio of 1 part curing agent to 32 parts base and a curing time of 6 h at 65°C, you will obtain a PDMS substrate with a Young's modulus of approx. 114 kPa (Tab. 1 in the main text).
- Cure the PDMS in an oven at 65°C for 24 hours.
- Remove the straws from the cured PDMS. Unscrew the mold frame and gently pull the substrate from the mold stamp. A finished PDMS substrate is shown in Fig. S15F. Note that a meniscus can form at the points where the straws are stuck in the PDMS, which means that the substrate bottom is not perfectly flat. Since this can cause problems for imaging, we recommend to carefully removing the menisci with a scalpel (Fig. S15G).


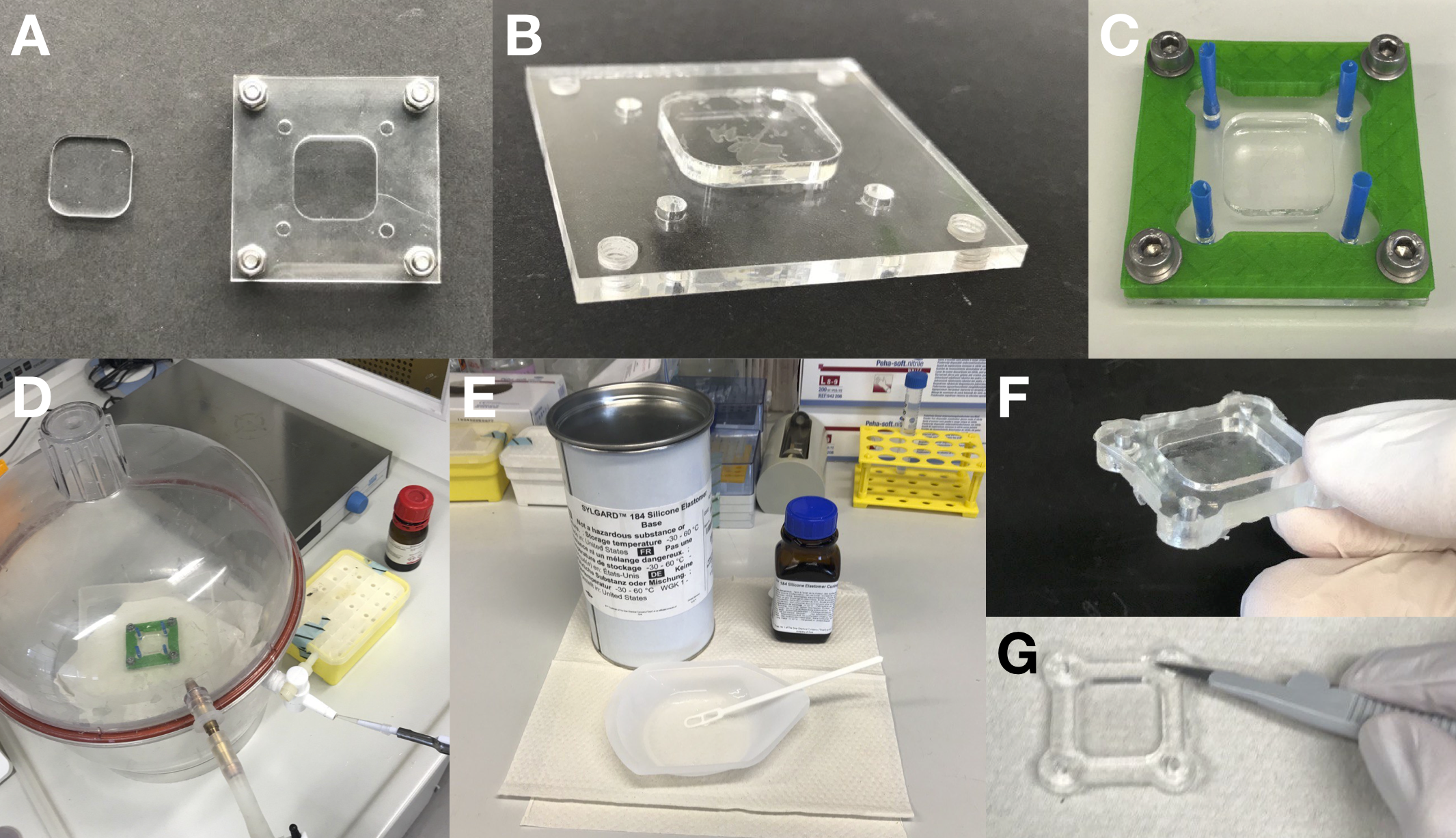


**Fig. S15: Assembly of the substrate molds and fabrication of PDMS cell substrates.**

# **S5 Operation instructions**

## **S5.1 Zero-point calibration**

**Tools:**

- **Caliper**

**Material:**

- **15 pin DSUB cable**
- **IEC power cable**
- **Peripheral devices for the Raspberry Pi**

In this section, we describe how to put the stretcher into operation and perform a zero-point calibration with the software *pyStretch*, i.e. set the point that is defined as 0% stretch.

- Connect the electronics and controller unit with the stretcher unit using a 15 pin DSUB cable of sufficient length. Power the control unit via a IEC power cable, connect a screen via HDMI, as well as a keyboard and a mouse via USB.
- Start the Raspberry Pi. Run the *PyStretch* software by double clicking on the desktop shortcut (see Supplementary Video 1). Click on "Execute" in the selection window.
- The *PyStretch* software consists of two windows. Click on the tab *"Calibration"* (marked with a red circle in Fig. S16A). In the Calibration window, click on the button labelled *"Calibrate to Standard Position"* (marked with a red circle in Fig. S16B).
- If the electronics and controller unit and the stretcher unit are correctly connected, the cell stretcher should start moving. The *"Calibrate to Standard Position"* button causes the mobile bar to move away from the stationary bar until it approaches the limit switch. The mobile bar should then move back towards the stationary bar and ideally stop after a distance of 135.9 mm. This corresponds to the distance at that the PDMS substrates can be inserted at exactly 0% stretch when the mountings are installed (see next section). If the stretcher creates high pitch noises while in use, you can apply precision engineering oil to the areas marked through red circles in Figure 14A while the stretcher moves.
- Measure the distance between sliding unit and fixed end with a caliper (Fig. S17B). If the distance is not 135.9 mm even after repeated measurements and restarting the calibration routine, you can change the distance the stretcher moves back after pressing the limit switch in the source code of the *PyStretch* software. For this, open the file /home/pi/py_stretch/motor_function.py with an editor (e.g. *Thonny*) and change the variable *mdist* in line 185 (Fig. S18). The value of the variable should correspond to the distance you measured between the mobile bar and the stationary bar (in units of mm).


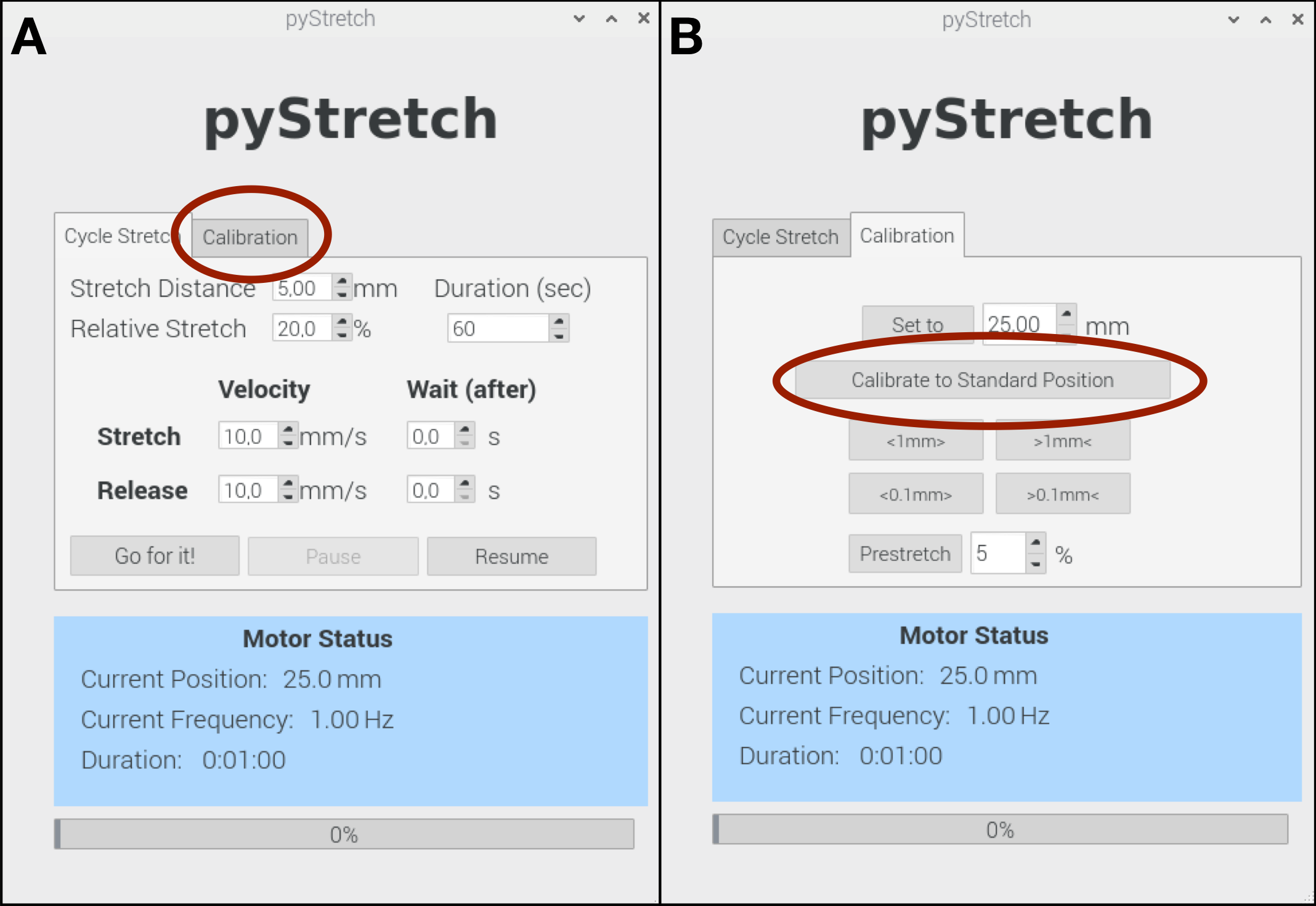


**Fig. S16: Graphical user interface of the *PyStretch* software.**


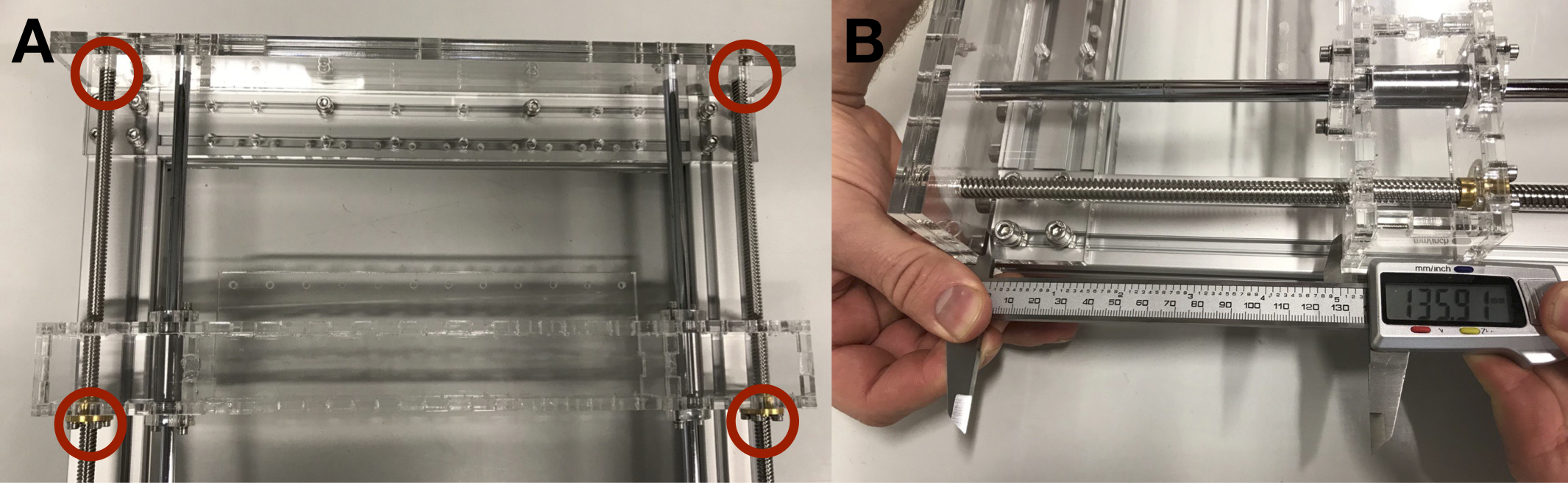


**Fig. S17: Zero-point calibration of the stretcher unit.**


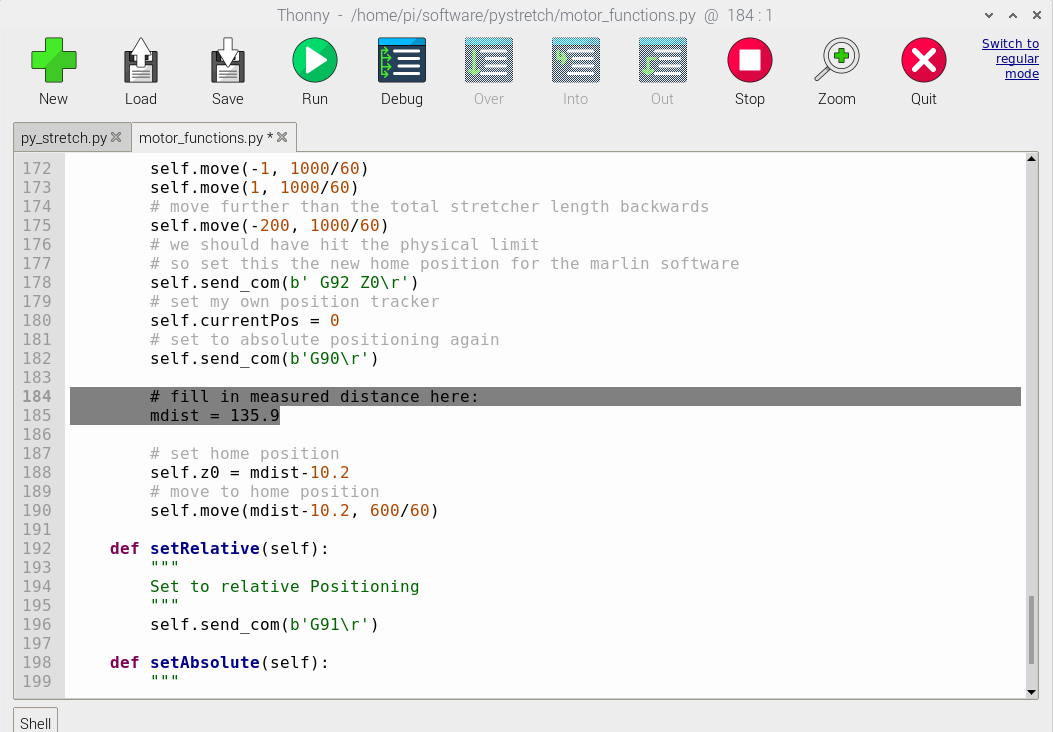


**Fig. S18: The distance between mobile bar and stationary bar that corresponds to the zero-point can be changed manually in the file *motor_functions.py*.**

## **S5.2 Install substrate mountings**

**Material:**

- **Acrylic glass parts: Mountings (12x)**
- **Screws: 24x (M3x12mm + spring washers + washers), 24x (M4x12mm + spring washers + washers)**
- Insert two M3x12mm screws in each acrylic glass mountings using washers and spring washers (Fig. S19A). These screws will serve as hooks to insert the PDMS substrates into.
- Perform the zero-point calibration as described in the previous section. Depending on how many samples you want to stretch simultaneously, you can now fix acrylic glass mountings in pairs opposite to each other as shown in Fig. S19B. Use two M4x12mm screws per mounting with spring washers and washers.

**
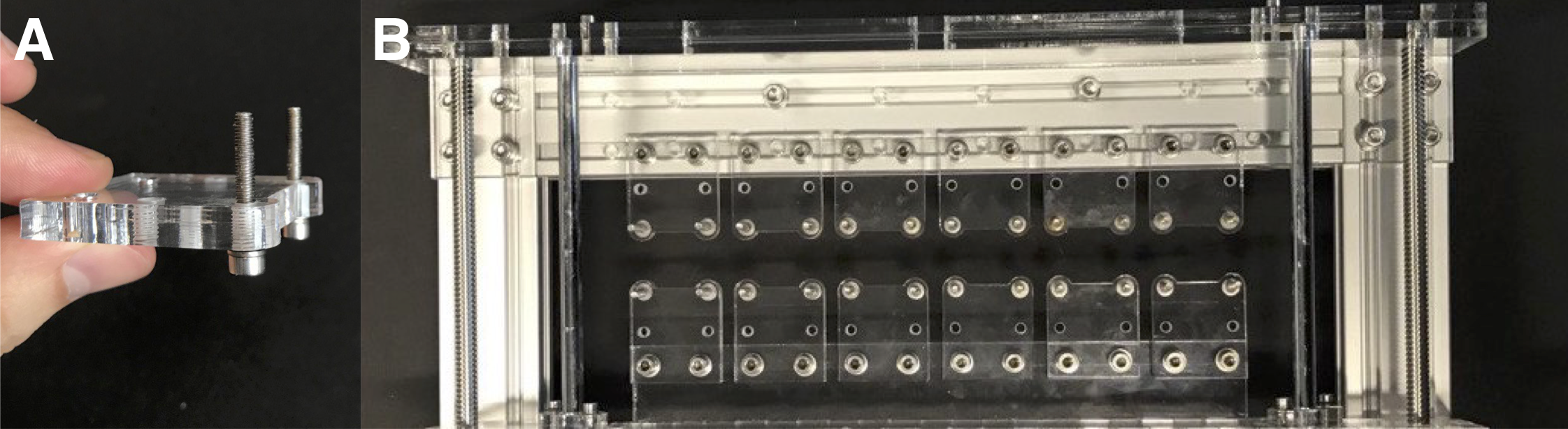
**

**Fig. S19: Substrate mountings attached to the rest of the stretcher unit.**

# **References**

[1] Raspberry Pi Imager. Available from: https://www.raspberrypi.org/blog/raspberry-pi-imager-imaging-utility, [Webpage] 2020

(cited 2020 14/05/2020).

[2] Arduino. Available from: https://www.arduino.cc/en/Main/AboutUs, [Webpage] 2020

(cited 2020 14/05/2020). Modified version with Anet A8 drivers available from: https://github.com/thijsk/Skynet3d/tree/master/arduino-1.8.0, [Webpage] 2020

(cited 2020 14/05/2020).

[3] Skynet3d. Adapted from: https://github.com/thijsk/Skynet3d, [Webpage] 2020

(cited 2020 14/05/2020).
