# Supplementary material for: A low-cost uniaxial cell stretcher for six parallel wells
Source: HardwareX. 2020 Dec 9;9:e00162. doi: 10.1016/j.ohx.2020.e00162 (PMC9041267; doi:10.1016/j.ohx.2020.e00162)
Supplement: Supplementary data 2 [file mmc2.docx]

**SUPPLEMENTAL DATA S6-12**

**Title:** A low-cost uniaxial cell stretcher for six parallel wells

**Authors:** Delf Kah^1,*^, Alexander Winterl^1^, Magdalena Přechová^2^, Ulrike Schöler^3,4^, Werner Schneider^1^, Oliver Friedrich^3,4^, Martin Gregor^2^, and Ben Fabry^1^

**Affiliations:** ^1^Biophysics Group, Department of Physics, Friedrich-Alexander University Erlangen-Nürnberg (FAU), Erlangen, Germany, ^2^Laboratory of Integrative Biology, Institute of Molecular Genetics of the Czech Academy of Sciences, Prague, Czech Republic, ^3^Institute of Medical Biotechnology, Department of Chemical and Biological Engineering, FAU, Erlangen, Germany, ^4^School in Advanced Optical Technologies, FAU, Erlangen, Germany, *Corresponding author.

**Contact email:** delf.kah@fau.de

In the following, we investigate the variability of the effective stretch across PDMS substrates presented in Fig. 8 in the main text. In summary, the effective stretch of the same PDMS substrate was highly reproducible for repeated measurements, it was not affected by the mounting position, and no wear or creep effects could be detected after one hour of static or cyclic stretch. Different PDMS substrates that had been produced following the same protocol, however, show slightly different effective stretch amplitudes with a coefficient of variation of 9%. The most likely explanation for this are slightly different bottom thicknesses of the individual substrates, which are due by the manufacturing process.

**Contents**

- **S6 Quantification of measured stretch**
- **S7 Measurement reproducibility**
- **S8 Relaxation**
- **S9 Positions**
- **S10 Static stretch**
- **S11 Cyclic stretch**
- **S12 Variability of substrates**

**S6 Quantification of measured stretch**

The following is a brief description of how the effective stretch on PDMS substrates applied with the six-well cell stretcher was quantified for Fig. 8 in the main text. For simplicity, Fig. S6 illustrates the stretch quantification with a sketch of a PDMS strip with only four marker points in a single column parallel to the stretch direction: P1, P2, P3, and P4 are the positions of four marker points on the unstretched PDMS substrate (Fig. S6A). The distance in stretch direction (i.e. neglecting differences in the position orthogonal to the stretch direction) of neighboring marker points are d_12_ (distance in stretch direction between P1 and P2), d_23_ (between P2 and P3), and d_34_ (between P3 and P4). After applying uniaxial stretch (e.g. 5 mm stretcher motion) to the PDMS substrate, the marker point positions change and the distances between marker points increase (Fig. S6B). The marker point positions of the stretched PDMS samples are P1’, P2’, P3’, and P4’ and the distances in stretch direction between neighboring marker points are d’_12_, d’_23_, and d’_34_. We quantify the measured stretch of the region between two marker points in stretch direction as the relative gain in distance through stretching (Fig. S6C). The relative line stretch (in percent) is, where d_12_ and d’_12_ have units of mm.


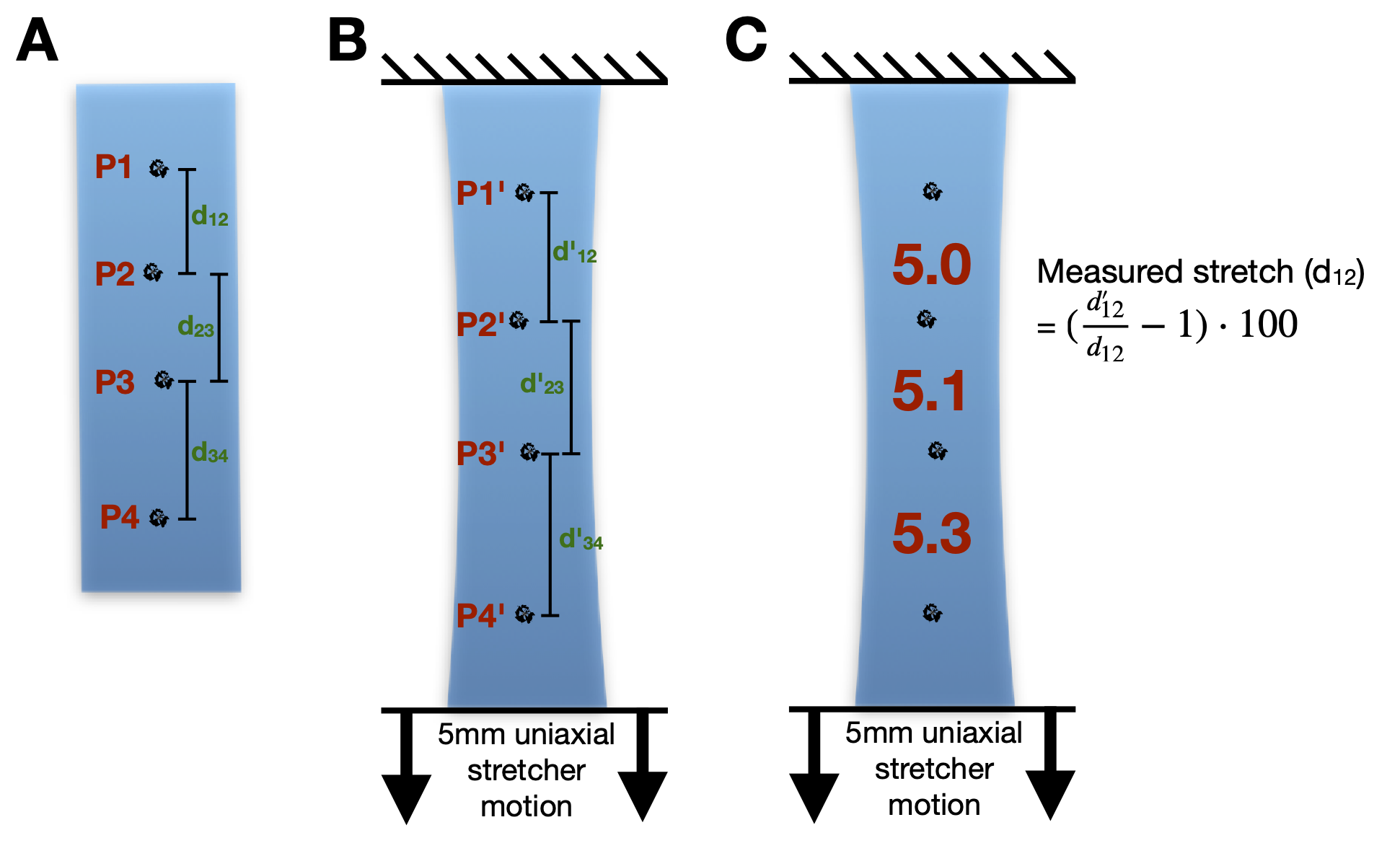


**Fig. S6.: Schematic illustration of the stretch quantification.** A: Unstretched PDMS sample with four marker points at positions P1-4 with distances in stretch direction d_12_, d_23_, and d_34_ between neighboring marker points. B: Uniaxial stretch of the same PDMS sample. The stretch results in changed marker point positions P’1-4, as well as elongated distances in stretch direction d’_12_, d’_23_, and d’_34_. C: The increase in distance between two marker points in percent is printed between the respective marker points, resulting in the form of representation in Fig.8C,F.

# **S7 Measurement reproducibility**

This section shows the reproducibility of the stretch verification measurement of Fig. 8. A PDMS substrate with a crosslinker ratio of 1:32 (curing time: 6 h) and a bottom thickness of approximately 500 µm (in the center of the substrate) was uniaxially stretched as described in Section 7 and the displacement of 36 painted marker points was photographed and evaluated. The effective stretch was quantified as described in Section S6. Note that in contrast to the measurements in the main text, here images were only taken at 0 mm, 2.5 mm, and 5 mm uniaxial stretch. Fig. S7 shows the evaluation of the effective substrate stretch analog to Fig. 8 in the main text for five repetitions. To avoid influences of the camera setup and substrate mounting on the measured stretch, the substrate was removed and remounted into the cell stretcher after each measurement (see Section 6.3), and the photo tripod was reconfigured. All measurements were performed with clamps. The PDMS substrate for this experiment was a different one than the one that was used for the measurements presented in the main text.

The data show that the measurement of the effective stretch is extremely reproducible. The values for the average effective stretch for the five repetitions were (5.28±0.58) %/mm, (5.28±0.58) %/mm, (5.27±0.60) %/mm, (5.30±0.59) %/mm, and (5.28±0.58) %/mm (mean±STD), respectively.


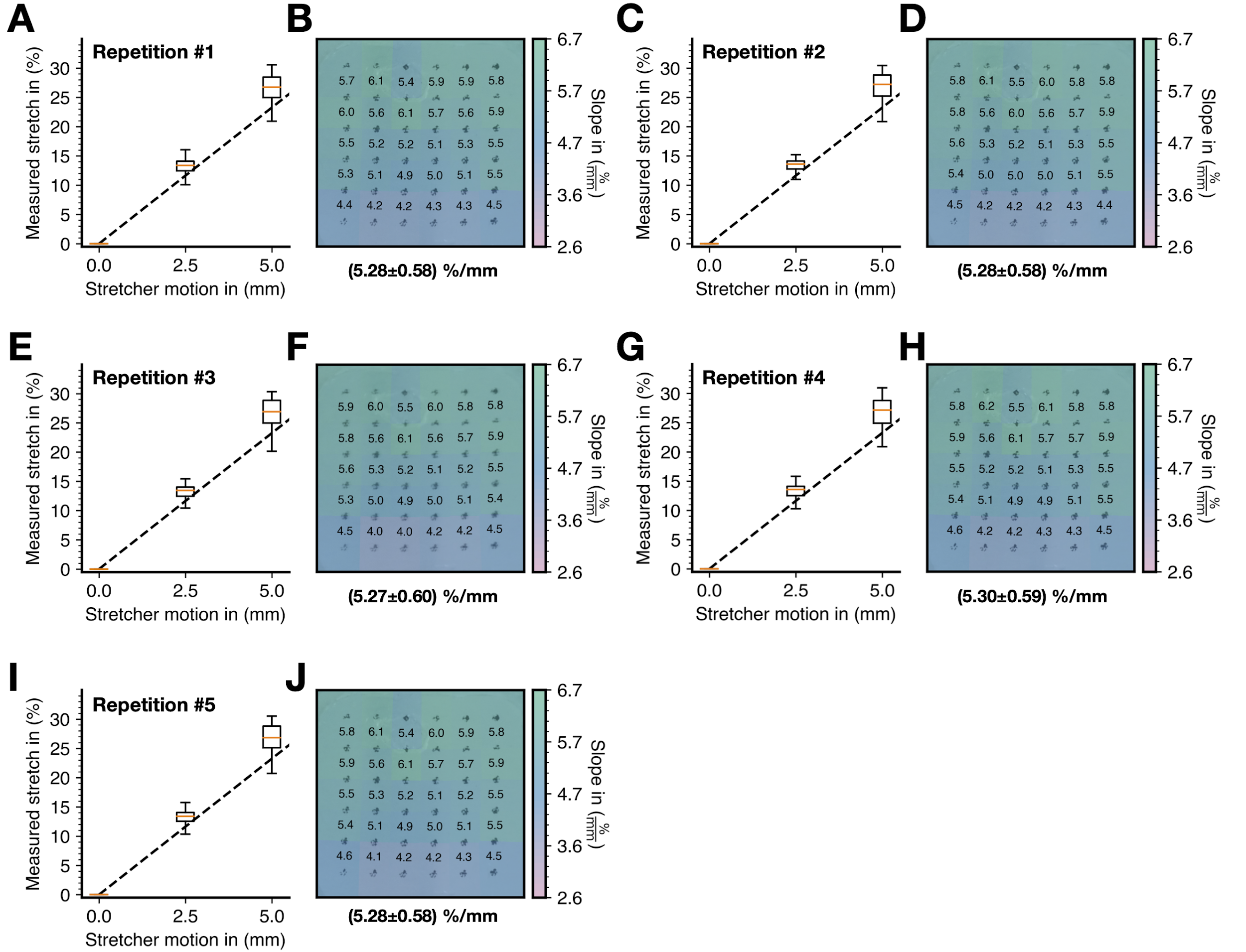


**Fig. S7.: Reproducibility of stretch measurement.** A: Measured relative stretch (in stretch direction) between two neighboring marker points for target stretches of 2.5 mm and 5 mm (median (orange line), 25% / 75% percentiles (boxes), 1.5 inter-quartile range (error bars)). Dashed line represents the target stretch assuming a baseline length of 21.5 mm. B: Spatial distribution of the effective stretch as determined by linear regression from the slope of the local stretch in percent versus mm stretcher motion. Bold numbers give the average effective stretch (mean±STD). C-J: Same as A&B, but for four repetitions.

# **S8 Relaxation**

This section shows the effective stretch of the same PDMS substrates from Fig.8, for a relaxation of applied uniaxial stretch from 5 mm back to 0 mm instead of a stretch from 0 mm to 5 mm. These relaxation measurements were performed immediately after the stretch measurements presented in Fig.8 with the intention to reveal potential hysteresis effects of the measured stretch due to wear effects of the PDMS. The uniaxial stretch with an amplitude of 5 mm was released in steps of 250 µm and the displacement of 36 painted marker points was photographed and evaluated. The measured stretch was quantified as described in Section S6.

The effective substrate stretch during relaxation was almost identical to the results of the stretch experiment in Fig.8, both for the setup with and without clamps. The average effective stretch for the substrate relaxation without clamps was (1.72±0.32) %/mm (Fig.S8A&B), which is comparable to the effective stretch of (1.68±0.33) %/mm presented in the main text (Fig.8A-C) (mean±STD). The average effective stretch for the substrate relaxation with clamps was (5.12±0.31) %/mm (Fig.S8C&D), which is comparable to the effective stretch of (5.13±0.30) %/mm presented in the main text (Fig.8D-F) (mean±STD). These measurements show that effective substrate stretch reaches the same amplitude, regardless of whether the stretch was achieved by expansion or relaxation, i.e. there are no hysteresis effects in the substrate stretch.


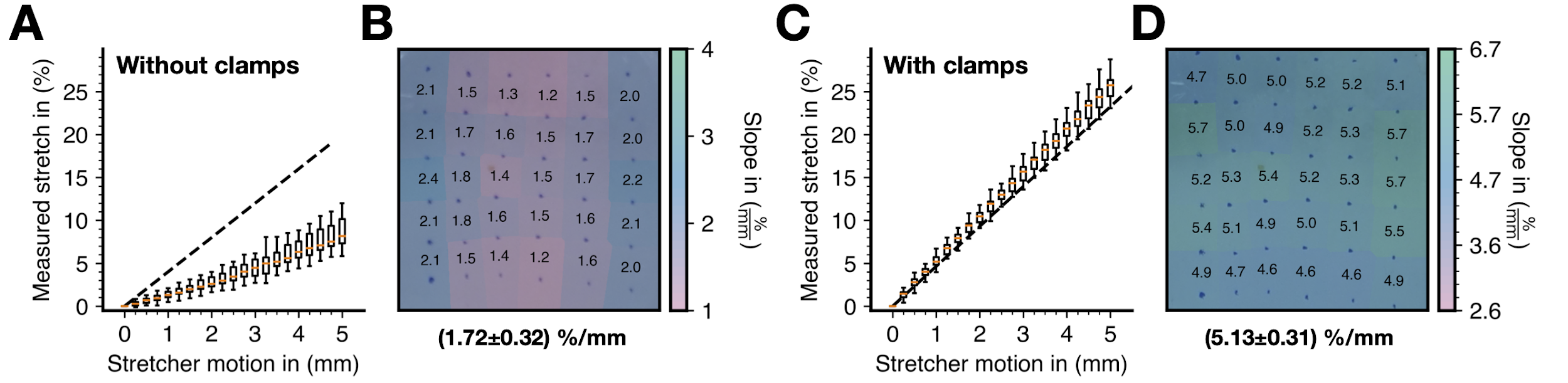


**Fig. S8.: Relaxation of stretched PDMS substrates.** A: Measured relative stretch (in stretch direction) between two neighboring marker points for target stretches between 5 mm and 0 mm in steps of 250 µm for a PDMS substrates mounted to the cell stretcher without clamps (median (orange line), 25% / 75% percentiles (boxes), 1.5 inter-quartile range (error bars)). Dashed line represents the target stretch assuming a baseline length of 25 mm. B: Spatial distribution of the effective stretch as determined by linear regression from the slope of the local stretch in percent versus mm stretcher motion. Bold numbers give the average effective stretch (mean±STD). C&D Same as A&B, but for a PDMS substrate mounted to the cell stretcher with clamps. Note that with clamps in place, a baseline length of 21.5 mm is considered.

# **S9 Positions**

This section shows the variability of the stretch verification measurement for a single PDMS substrate that was stretched at all six different positions of the cell stretcher (see Fig. 2F). For this, a PDMS substrate with a crosslinker ratio of 1:32 (curing time: 6 h) and a bottom thickness of approximately 500 µm (in the center of the substrate) was uniaxially stretched as described in Section 7 and the displacement of 36 painted marker points was photographed and evaluated. The measured stretch was quantified as described in Section S6. Note that in contrast to the measurements in the main text, here images were only taken at 0 mm, 2.5 mm, and 5 mm uniaxial stretch. The PDMS substrate for this experiment was yet a different one than the one that was used for the measurements presented in Sections S7&8.

Fig. S9 shows the evaluation of the measured substrate stretch analog to Fig. 8 in the main text. Positions 1-6 denote the different positions in which a PDMS substrate can be mounted into the cell stretcher, where position 1 is the leftmost position and position 6 is the rightmost position when positioning the cell stretcher as seen in Fig. 2F. The measured effective stretch on the substrate showed only marginal differences when measured at the six different positions. More specifically, the measured stretch was (4.92±0.48) %/mm at position 1, (4.87±0.44) %/mm at position 2, (4.89±0.44) %/mm at position 3, (4.83±0.40) %/mm at position 4, (4.84±0.42) %/mm at position 5, and (4.88±0.45) %/mm at position 6 (mean±STD).


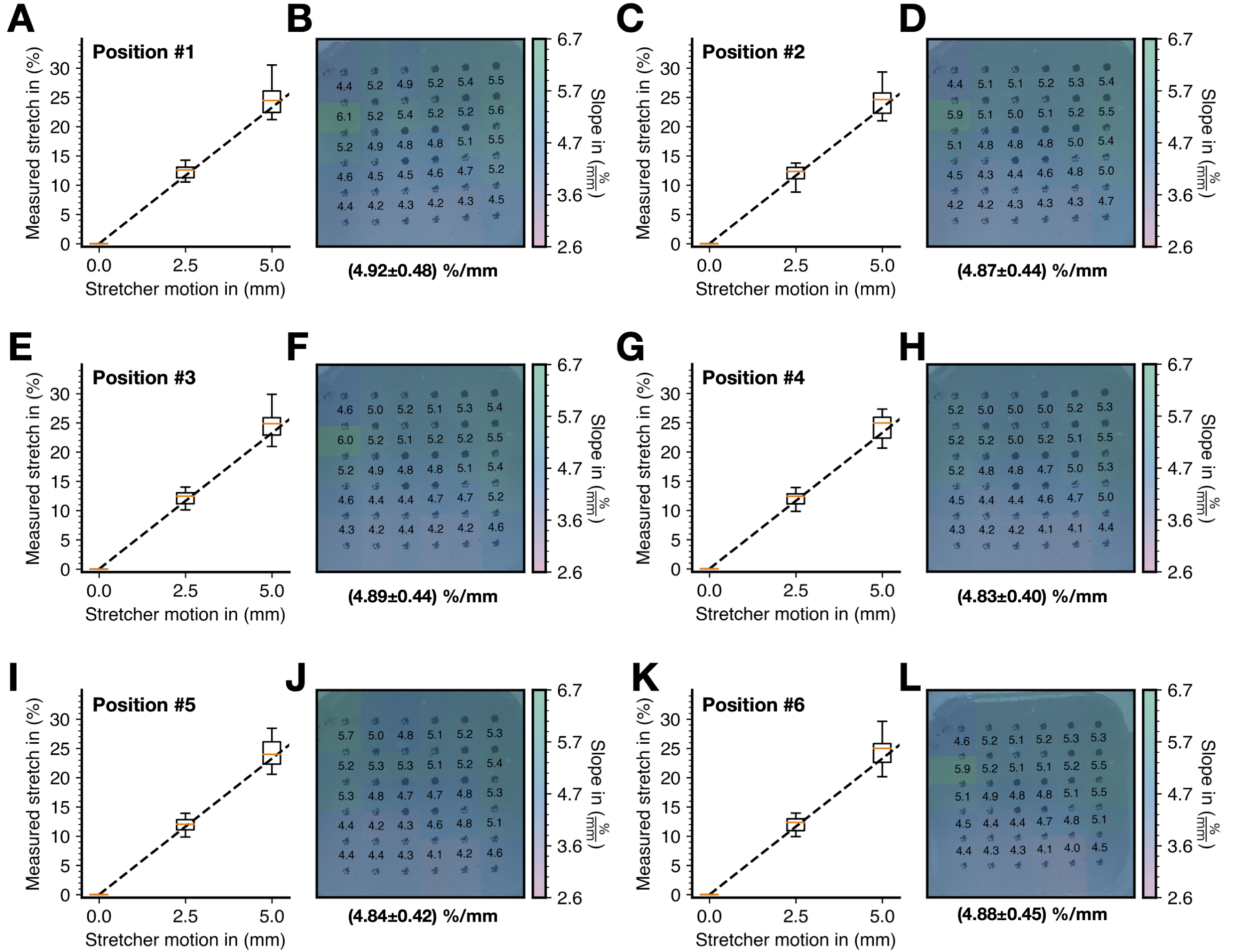


**Fig. S9.: Stretch measurement at different positions.** A: Measured relative stretch (in stretch direction) between two neighboring marker points for target stretches of 2.5 mm and 5 mm (median (orange line), 25% / 75% percentiles (boxes), 1.5 inter-quartile range (error bars)). Dashed line represents the target stretch assuming a baseline length of 21.5 mm. B: Spatial distribution of the effective stretch as determined by linear regression from the slope of the local stretch in percent versus mm stretcher motion. Bold numbers give the average effective stretch (mean±STD). C-L: Same as A&B, but for five further measurements of the effective stretch of the same substrate when mounted at different positions of the six-well cell stretcher.

**S10 Static stretch**

This section shows the variability of the stretch verification measurement for a single PDMS substrate that was kept at a static stretch of 5 mm and imaged five times over the course of one hour. The purpose of this experiment was to investigate whether the measured stretch changes over time due to wear effects of the PDMS. For this, a PDMS substrate with a crosslinker ratio of 1:32 (curing time: 6 h) and a bottom thickness of approximately 500 µm (in the center of the substrate) was uniaxially stretched as described in Section 7 and the displacement of 36 painted marker points was photographed and evaluated. The measured stretch was quantified as described in Section S6 immediately after applying the stretch, as well as 1 min, 5 min, 10 min, and 60 min afterwards. Note that in contrast to the measurements in the main text, here a single image was taken at 0 mm uniaxial stretch, as well as five images at 5 mm uniaxial after the respective stretch duration. The PDMS substrate for this experiment was yet a different one than the one that was used for the measurements presented in Sections S7-9.

Fig. S10 shows the evaluation of the measured substrate stretch analog to Fig. 8 in the main text. The measured effective stretch on the substrate showed only marginal differences when measured immediately, as well as after 1, 5, 10, and 60 min of applied static stretch. More specifically, the measured stretch was (4.65±0.31) %/mm immediately after applying the stretch, (4.63±0.31) %/mm after 1 min, (4.65±0.31) %/mm after 5 min, (4.65±0.29) %/mm after 10 min, and (4.66±0.29) %/mm after 60 min (mean±STD). There was no evident wear effect of the PDMS after one hour of constant static stretch with an amplitude of 5 mm.


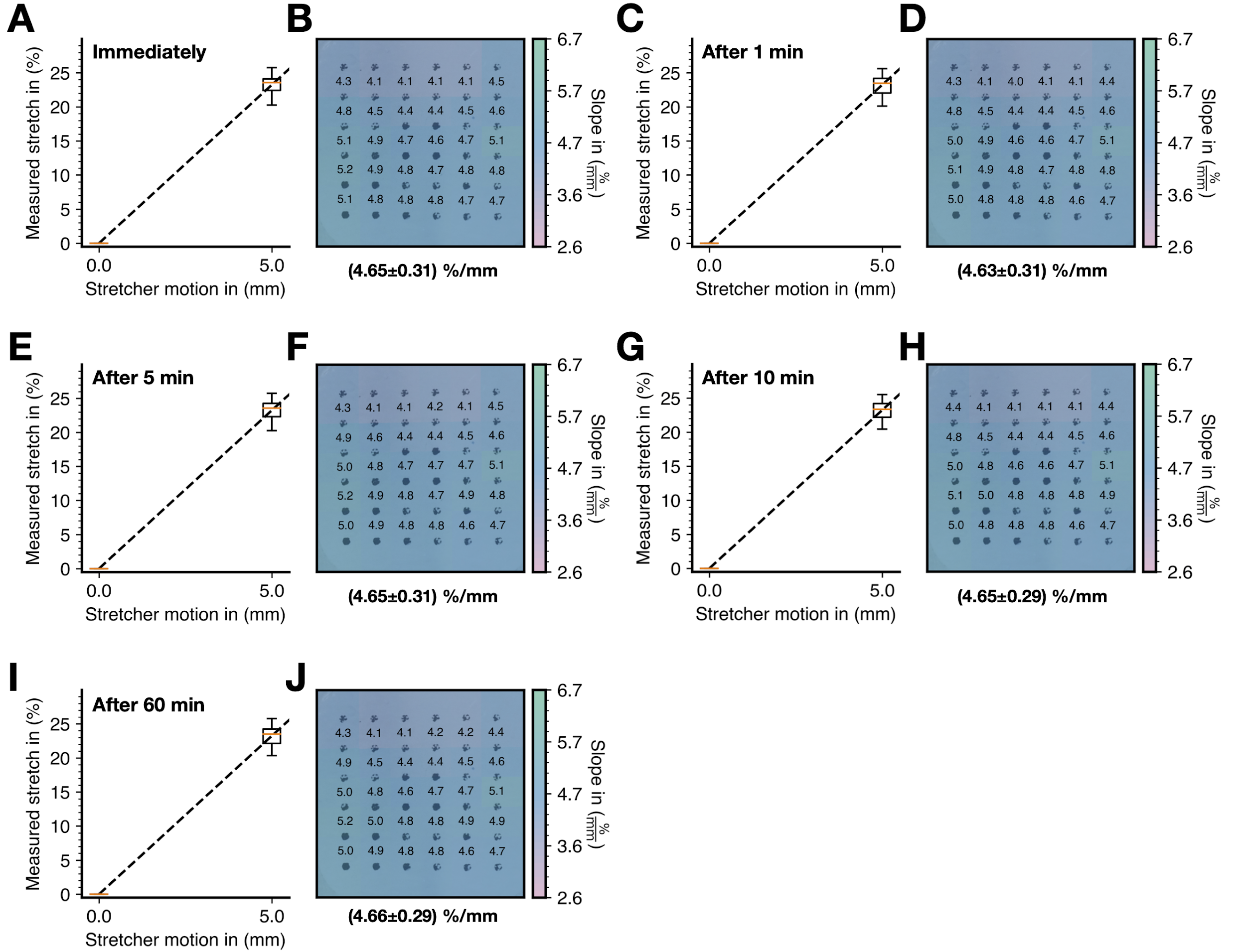


**Fig. S10.: Stretch measurement for different durations of applied static stretch.** A: Measured relative stretch (in stretch direction) between two neighboring marker points immediately after applying static stretch with an amplitude of 5 mm compared to an image taken before applying static stretch (median (orange line), 25% / 75% percentiles (boxes), 1.5 inter-quartile range (error bars)). Dashed line represents the target stretch assuming a baseline length of 21.5 mm. B: Spatial distribution of the effective stretch as determined by linear regression from the slope of the local stretch in percent versus mm stretcher motion. Bold numbers give the average effective stretch (mean±STD). C-J: Same as A&B, but for images taken after 1, 5, 10, and 60 min of continuous static stretch.

**S11 Cyclic stretch**

This section shows the variability of the stretch verification measurement for a single PDMS substrate that was exposed to 60 min of cyclic stretch with an amplitude of 5 mm and a frequency of 0.5 Hz. The purpose of this experiment was to investigate whether the measured stretch changes over time due to wear effects of the PDMS. For this, a PDMS substrate with a crosslinker ratio of 1:32 (curing time: 6 h) and a bottom thickness of approximately 500 µm (in the center of the substrate) was uniaxially stretched as described in Section 7 and the displacement of 36 painted marker points was photographed and evaluated before and after 60 min of applied cyclic stretch. The measured stretch was quantified as described in Section S7. Note that in contrast to the measurements in the main text, here images were only taken at 0 mm, 2.5 mm, and 5 mm uniaxial stretch. The PDMS substrate for this experiment was yet a different one than the one that was used for the measurements presented in Sections S7-10.

Fig. S10 shows the evaluation of the measured substrate stretch analog to Fig. 8 in the main text. The measured effective stretch on the substrate showed only marginal differences when measured before and after 60 min applied cyclic stretch. More specifically, the measured stretch was (4.86±0.58) %/mm before and (4.91±0.56) %/mm after 60 min (mean±STD). There was no evident wear out effect of the PDMS after one hour of cyclic stretch with an amplitude of 5 mm and a frequency of 0.5 Hz.


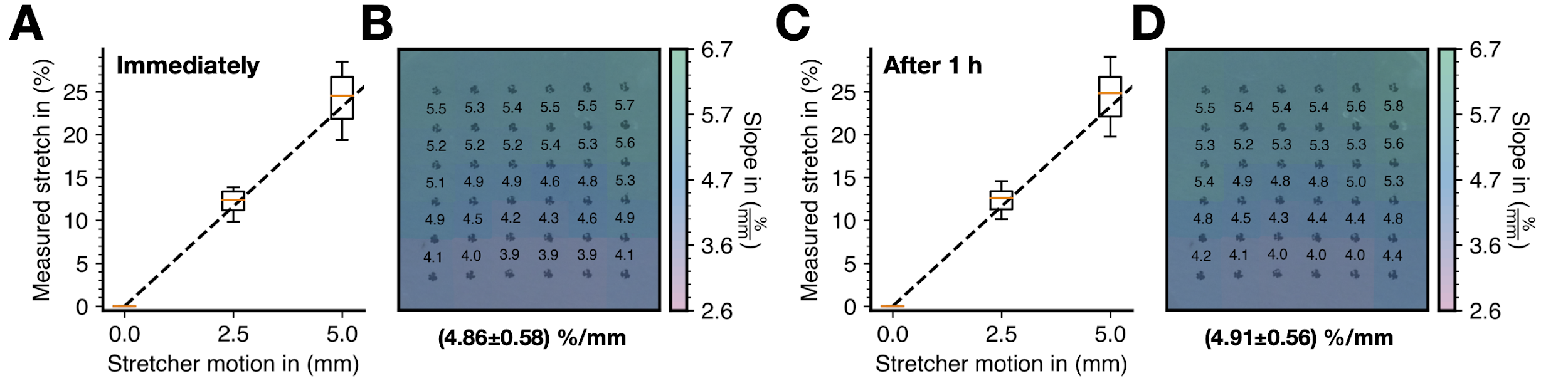


**Fig. S11.: Stretch measurement before and after 1 h of cyclic stretch.** A: Measured relative stretch (in stretch direction) between two neighboring marker points for target stretches of 2.5 mm and 5 mm (median (orange line), 25% / 75% percentiles (boxes), 1.5 inter-quartile range (error bars)). Dashed line represents the target stretch assuming a baseline length of 21.5 mm. B: Spatial distribution of the effective stretch as determined by linear regression from the slope of the local stretch in percent versus mm stretcher motion. Bold numbers give the average effective stretch (mean±STD). C&D: Same as A&B, but for a repeated measurement after 1 h of applied cyclic stretch.

**S12 Variability of substrates**

Sections S7-S11 show that the measurement of substrate stretch as described in Section 7 yields comparable results for repeated measurements of the same substrate at the same stretch position (Section S7), for comparing stretching and relaxing of the same substrate (Section S8), for measurements of the same substrate at different stretch positions (S9), for repeated measurements of the same substrate at different time points when kept at a static stretch for one hour (S10), and for the same substrate before and after one hour of cyclic stretch (S11). Because all of these experiments were performed with different PDMS substrates that were fabricated following the same protocol (each with a crosslinker ratio of 1:32 and a curing time of 6 h), they can be used to quantify the variability of the measured stretch of separately casted PDMS substrates. For this, we averaged the measured stretch of all distances of neighboring marker points (see Section S6) of all repetitions of a given experiment. Note that we compare only the experiments where PDMS substrates were stretched using clamps (i.e. we exclude the measurements from Fig. S8.1). The results are presented in Tab. S12. In contrast to repeated measurements of identical samples, comparing different PDMS substrates yields a coefficient of variation of 9%. The most likely explanation for this are slightly different bottom thicknesses of the individual substrates, which are due by the manufacturing process. The average measured stretch is (4.96±0.46) %/mm (mean±STD, determined through propagation of error).

**Tab. S12.: Comparison of average measured stretch of different PDMS substrates.** The measured stretch of five individual PDMS substrates that were fabricated following the same protocol (1:32 crosslinker ratio, 6 h curing time) is given as mean values and standard deviation.

| **Experiment** | **Mean (%/mm)** | **STD (%/mm)** |
| --- | --- | --- |
| S7 | 5.28 | 0.59 |
| S8* | 5.12 | 0.31 |
| S9 | 4.87 | 0.44 |
| S10 | 4.65 | 0.31 |
| S11 | 4.88 | 0.57 |

*also including the data from Fig. 8 D-F in the main text
